# Assessing trends and density of bird species in bottomland hardwood forests and riparian forests using simulation and sample size optimization for surveys

**DOI:** 10.1038/s41598-025-91804-4

**Published:** 2025-02-28

**Authors:** David R. Stewart, Steven E. Sesnie, Paige Schmidt, David Londe, Matthew J. Butler, Grant M. Harris, John Stephens, James M. Mueller

**Affiliations:** 1 Division of Biological Sciences, U.S. Fish and Wildlife Service, Albuquerque, NM 87103 USA; 2 U.S. Fish and Wildlife Service, 9014 East 21st St., Tulsa, OK 74129 USA; 3 Wichita Mountains Wildlife Refuge, U.S. Fish and Wildlife Service, 32 Headquarters Road, Indiahoma, OK 73552 USA; 4 Caddo Lake National Wildlife Refuge, U.S. Fish and Wildlife Service, 15600 FM 134, Karnack, TX 75661 USA; 5 Balcones Canyonlands National Wildlife Refuge, U.S. Fish and Wildlife Service, 24518 FM 1431, Marble Falls, TX 78654 USA

**Keywords:** Avian surveys, Bottomland hardwood and riparian forests, Conservation monitoring, Multispecies surveys, Point counts, Ecology, Ecology

## Abstract

The decline of neotropical migratory birds in North America is closely tied to habitat loss, including the degradation of bottomland hardwood and riparian forests, which provide essential habitats for numerous species. To address this, the U.S. Fish and Wildlife Service conducts bird surveys to monitor restoration efforts and evaluate conservation outcomes. This study assessed avian surveys from three National Wildlife Refuges in Texas and Oklahoma, using simulations, field data, and literature to evaluate current sampling protocols. Our findings revealed that achieving acceptable precision in bird density estimates (coefficient of variation: 0.15, 0.25) often requires more than 200 bird point counts, depending on the species and study area. While data aggregation across sites and years improved precision, it masked local trends critical for refuge-specific management. Imprecise results, particularly for rare species, underscored the need for improved protocols, such as repeat visits within a year, targeted sampling for priority species, and adaptive designs incorporating forest composition and structure data. These adjustments would enhance the precision of multispecies surveys, making them more effective for detecting changes in habitat quality. This study provides actionable recommendations to support service-wide efforts in strategic, data-driven monitoring and long-term conservation planning for neotropical migratory birds.

## Introduction

Bottomland hardwood and riparian forests, shaped by their dynamic hydrology and fertile, wet soils, stand as critical habitat for avian biodiversity throughout their breeding, nonbreeding, and migration periods^1,2^. These ecosystems, once defined by natural flooding cycles that maintained their structure and diversity, have been profoundly altered by anthropogenic changes such as flood control measures, disrupting ecological processes and reducing habitat quality for nesting birds^3,4^. Alarmingly, in regions like the Lower Mississippi Alluvial Valley, less than one-fifth of the pre-Euroamerican bottomland hardwood and riparian forests remain^5^. The extensive loss of these critical ecosystems has emerged as a significant conservation concern, threatening the birds and other wildlife that depend on them^3,4,6^.

To address habitat loss and decline in bird populations dependent on bottomland hardwood and riparian forests, conservation planners emphasize the importance of habitat restoration^7^. The composition and structure of these managed forests can differ based on geographic location and the timing of restoration efforts, directly influencing the bird communities they support^8^^,^^9^. As such, monitoring bird population trends has become a key metric for evaluating the success of restoration initiatives and ensuring that management aligns with the habitat needs of target bird species^10^. However, survey results are often affected by factors that can introduce bias and obscure true population trends, including survey effort, site selection, climatic variability, and habitat conditions both within and beyond the study area (e.g., edge effects, conditions in wintering grounds). Identifying and addressing these sources of bias is essential to refining survey methodologies and designing effective long-term monitoring programs.

Our primary objective was to assess whether current survey methods are sufficient for estimating population trends and densities, while addressing biases and limitations inherent in multispecies monitoring. We also assessed the efficacy of these methods against the optimal number of point counts and the standards observed in the literature, aiming to determine how well existing surveys provide reliable status and trend estimates for species. This analysis is particularly critical for designing surveys that effectively account for the challenges of detecting rare and less detectable species. Historically, bottomland hardwood and riparian forests were exploited for timber products, oil and gas extraction, agriculture, and even military operations, leaving significant ecological impacts. Some of these areas have since been acquired by the USFWS and are now managed as part of the National Wildlife Refuge (NWR) System, where consumptive activities have diminished or ceased. Land managers at these refuges strive to restore and maintain bottomland hardwood and riparian forests within established desired forest conditions, while adhering to landscape and site-scale environments (Table 1; LMVJV Forest Resource Conservation Working Group^8^). To evaluate the effectiveness of these restoration efforts, annual breeding bird surveys were implemented to monitor population trends of bottomland hardwood and riparian forest bird species. However, these surveys were initiated without a critical assessment of their adequacy for estimating trends of focal species or identifying potential sources of bias. This gap underscores the need for a comprehensive evaluation to ensure that monitoring programs effectively align with management goals and address the complexities of multispecies conservation.

**Table 1:** Desired forest conditions for bottomland hardwood and riparian forests, as defined by management objectives at three National Wildlife Refuges (NWR) in Oklahoma and Texas, USA and the Lower Mississippi Valley Joint Venture [LMVJV] Forest Resource Conservation Working Group (2007).

Forest variables	Desired forest structure			
	Little River NWR	Caddo Lake NWR	Little sandy NWR	LMVJV
Overstory canopy cover	50–70%	60–70%	60–70%	60–70%
Basal area	14–21 m^2^/ha	9–18 m^2^/ha	9–18 m^2^/ha	14–16 m^2^/ha
Emergent crowns	5–15%	–	–	–
Midstory cover	< 20% or > 50%	–	–	25–40%
Understory cover	40–60%	–	–	25–40%
Ground cover	20–50%	> 40%	> 40%	–
Vines in all 4 canopies	70%	–	–	–
Giant cane	Present at 20% of plots	–	–	–

These targets are used to guide restoration efforts and provide context for evaluating bird population monitoring results in this study.

Designing adequate and efficient surveys to monitor bird populations within bottomland hardwood and riparian forests is challenging due to the diverse habitat preferences of species targeted for management. For instance, the abundance of many forest birds, such as the Swainson’s Warbler (*Limnothlypis swainsonii*) and the Kentucky Warbler (*Geothlypis formosa*), is closely tied to vegetation composition and structure^11^. Kentucky Warblers thrive in areas near streams with dense understory thickets and expansive foliage layers (McShea et al. 1995). In contrast, Swainson’s Warbler occupy canebrakes (*Arundinaria gigantea*) interspersed with canopy gaps and also favor disturbance gaps densely population with saplings, shrubs, and vines for nesting^12^^,^^13^^,^^14^. To achieve management objectives, survey designs must ensure sufficient sampling effort across all relevant habitats, capture the full spectrum of species-specific preferences within these complex ecosystems.

In light of the variability in habitat use and the challenges of designing efficient surveys, a key objective of this study is to determine the survey effort (i.e., the number of point counts by species and study area) required to achieve reliable density estimates with sufficient precision to support both research and management goals. Specifically, we aimed to achieve coefficients of variation (CV) of 0.15 for research-focused surveys requiring high precision and 0.25 for monitoring-focused surveys aimed at informing management actions^15^. These thresholds are widely used in ecological monitoring and reflect levels of precision needed for detecting meaningful trends in population densities and evaluating the effects of habitat management interventions^15–17^.

Distance-sampling and time-removal models are among the most widely used methods for estimating the abundance of avian species^18–20^. These methods are efficient, requiring observers to record both the time interval and estimated distance to each bird upon first detection during a survey, making them particularly well-suited for large-scale, long-term avian monitoring programs. Despite their efficiency, uncertainty persists regarding the ability of these methods to produce unbiased density estimates for less common and less detectable bird species, which are often the primary focus of conservation efforts. Moreover, our literature review highlights a significant knowledge gap in understanding the precision and reliability of these methods for uncommon species in bottomland hardwood and riparian forests. This concern is especially relevant when survey efforts remain consistent regardless of species distributions and undefined precision goals. If these methods prove data-intensive, requiring large sample sizes (e.g., numerous point counts) to achieve sufficient precision for detecting trends, biologists may need to reconsider their approach and resources. This could involve reallocating resources to increase sample sizes or adopting alternative monitoring techniques.

**Fig. 1:**
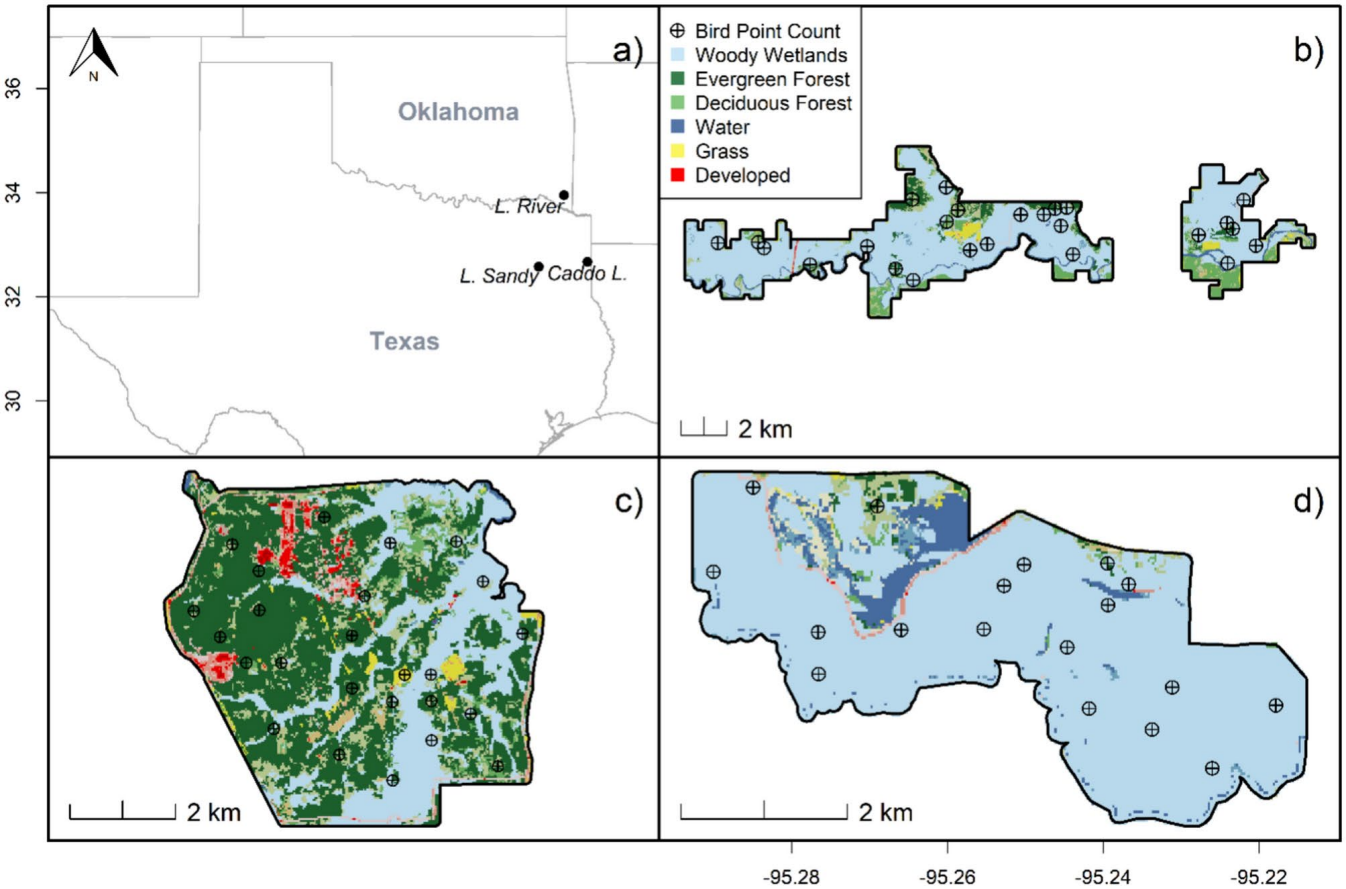
Map of Oklahoma and Texas, USA (**a**) showing three U.S. Fish and Wildlife Service National Wildlife Refuges we used in a survey of abundance of bottomland hardwood and riparian forest bird species, May to June 2008–2020. Bird point locations at (**b**) Little River National Wildlife Refuge, (**c**) Caddo Lake National Wildlife Refuge, and (**d**) Little Sandy National Wildlife Refuge. Vegetation types come from the 2019 National Land Cover Database layer for Texas and Oklahoma. The map was generated in program R^70^.

We used simulation to test how bias and precision in bird density estimates from point counts are influenced by factors such as probability of availability (i.e., the likelihood that a bird is present and available for detection during a survey) and presence (i.e., if the bird is actually there to be detected or not), especially for rare and less detectable species, such as the Swainson’s Warbler. This approach allowed us to explore how the number of bird point counts and the frequency of repeat visits affect estimation accuracy under varying conditions. By building on this model, we predicted the population trends of bottomland hardwood and riparian forest birds inhabiting some of the last forested remnants in Oklahoma and Texas, USA, namely at Little River NWR, Caddo Lake NWR, Little Sandy NWR, and all three refuges combined (Fig. 1). We examined how the results could inform management decisions by determining whether survey adjustments are needed, assessing the feasibility of such adjustments, and proposing alternative approaches that provide useful data for managers while being more logistically feasible. Additionally, we assessed the optimal number of point counts that would be needed to produce species-level population assessments for a given level of precision. To contextualize our findings, we conducted a review of studies on bird species in bottomland hardwood and riparian forests to summarize the typical range and methodologies of point counts employed in the field. This comparison helps evaluate how our survey design aligns with best practices and provides insights to optimize monitoring strategies for multispecies surveys in similar ecosystems. Our work contributes to the development of more efficient, practical, and impactful monitoring strategies that balance precision, feasibility, and resource constraints. Our results enhance conservation outcomes for birds inhabiting bottomland hardwood and riparian forests and similar ecosystems.

### Study area

We conducted our study on three National Wildlife Refuges (NWR) within the South Central Plains ecoregion^21^ in Oklahoma and Texas (Fig. 1). This region represents a westward extension from the Mississippi River of pine, mixed hardwood, and bottomland hardwood and riparian forests^22^. Little River NWR (5753 ha), encompassing mixed hardwood and bottomland hardwood and riparian forests in southern Oklahoma, occurs in McCurtain County. Caddo Lake NWR (3440 ha), within Harrison County, Texas, was transferred to USFWS in October 2000 from the Department of Defense, formerly the Longhorn Army Ammunition Plant. Little Sandy NWR (1539 ha) sits on the northern bank of the Sabine River in southern Wood County, Texas. Dominant overstory species included sweetgum (*Liquidambar styraciflua*), blackgum (*Nyssa sylvatica*), loblolly pine (*Pinus taeda*), overcup oak (*Quercus lyrata*), cherrybark oak (*Q. pagoda*), swamp chestnut oak (*Q. michauxii*), water oak (*Q. nigra*), willow oak (*Q. phellos*), bald cypress (*Taxodium distichum*), American elm (*Ulmus americana*), and cedar elm (*U. crassifolia*). The dominant midstory species were common buttonbush (*Cephalanthus occidentalis*), swamp privet (*Forestiera ligustrina*), green ash (*Fraxinus pennsylvanica*), American holly (*Ilex opaca*), southern sugar maple (*Acer floridanum*), and water elm (*Planera aquatica*).

## Results

### Simulation results

**Fig. 2:**
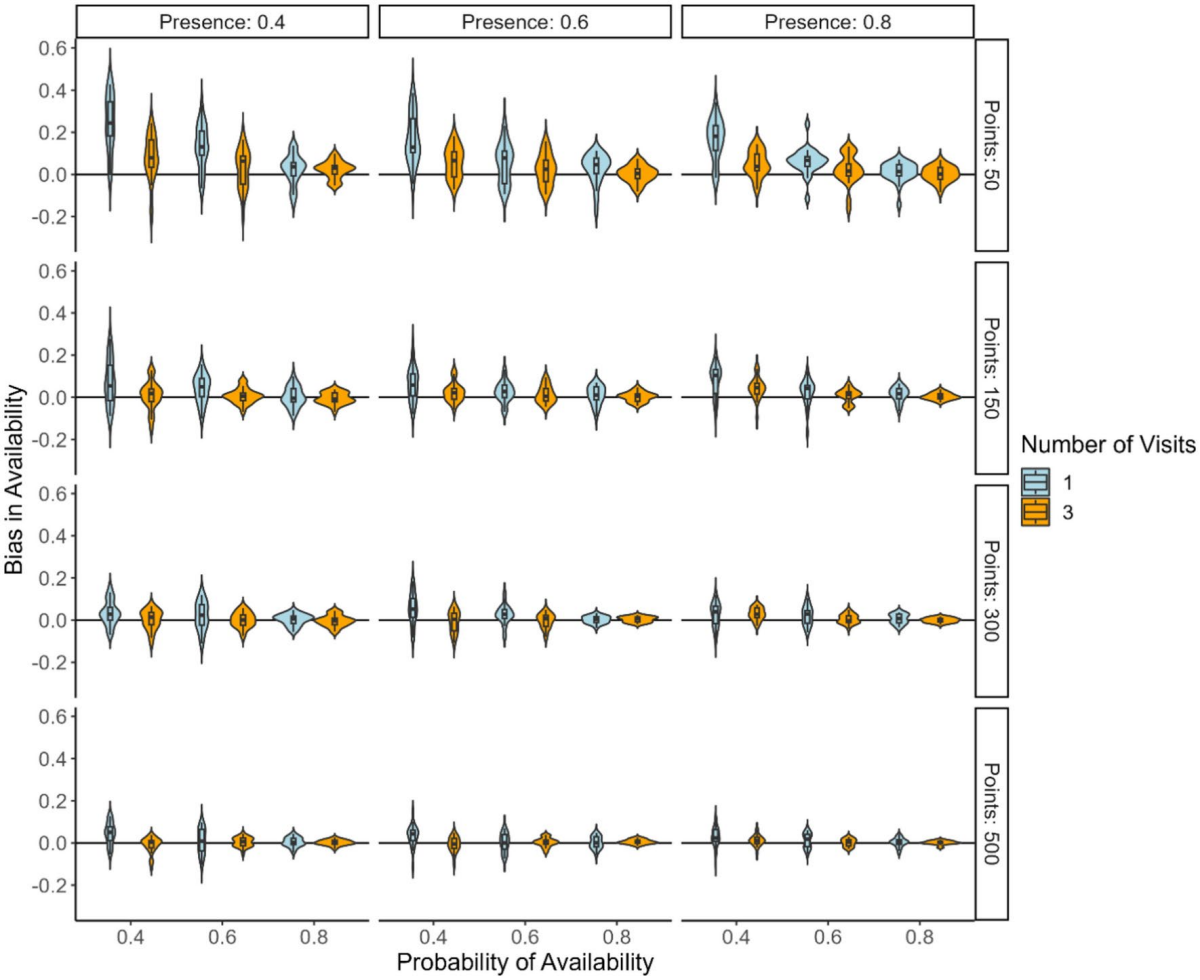
Bias in estimating the probability of availability summarized as violin plots from multiple simulated datasets under varied condition. The scenarios are differentiated by the probability of presence (0.4, 0.6, 0.8) and the number of repeat visits (1 and 3) in a single season, with further categorization by different numbers of bird point counts (50, 150, 300, and 500). Values represent the posterior model minus true value.

Bias in the probability of availability across all presence levels was generally small and clustered around zero (Fig. 2). Most bias occurred with species having low presence (0.4), where biases were more varied for single and three visit surveys. For a moderate presence (0.6), single visit biases show greater variance, which is reduced with three visits. High presence (0.8) scenarios display tightly clustered biases for both one and three visit surveys, indicating consistent unbiased estimation irrespective of the number of visits. The number of point counts (50, 150, 300, and 500), represented in Fig. 2, does not appear to substantially affect the bias, as the distributions for different point values generally overlap.

**Fig. 3:**
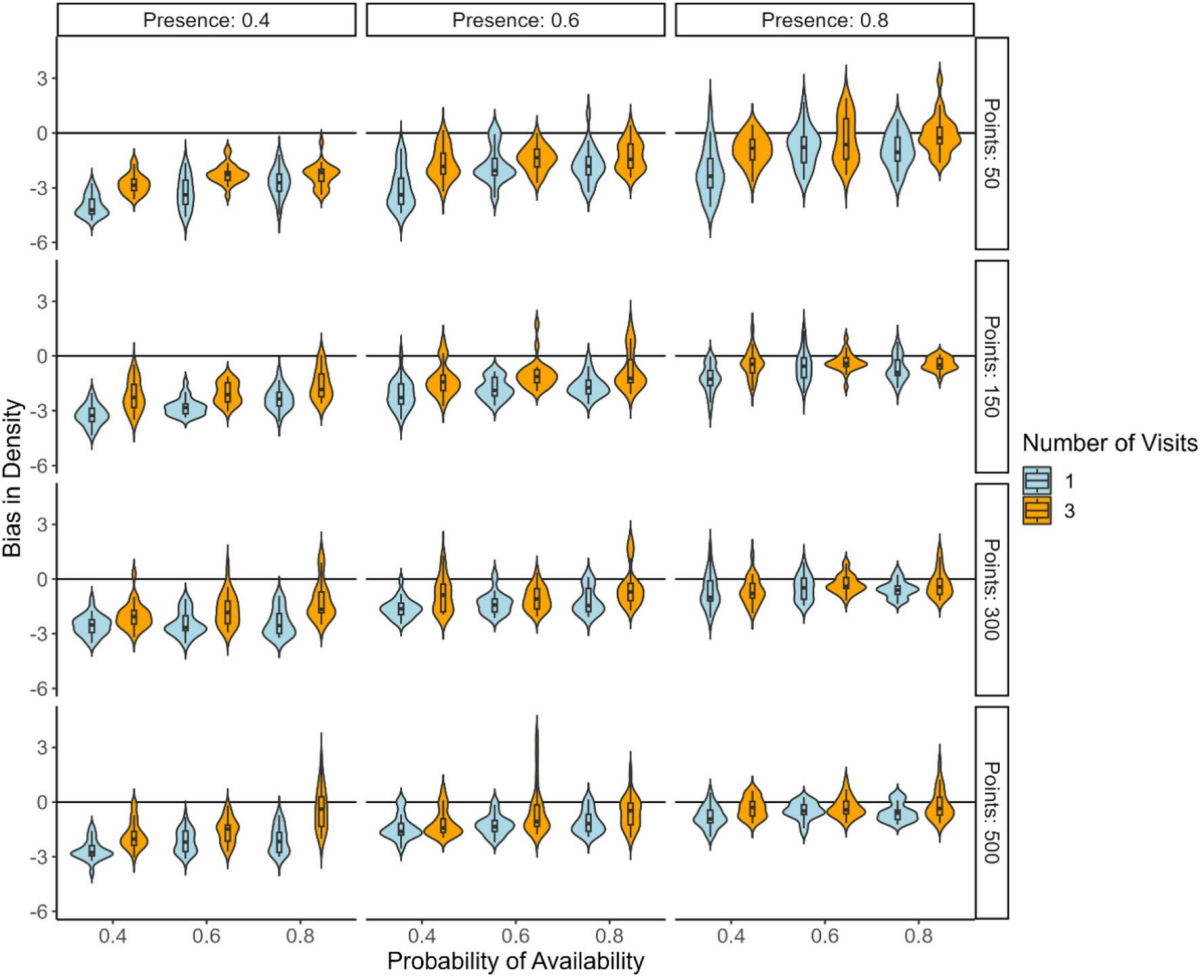
Violin plots summarizing the bias in estimating density from multiple simulated datasets under varied condition. The scenarios are differentiated by the probability of presence (0.4, 0.6, 0.8) and the number of repeat visits (1 and 3), with further categorization by different numbers of bird point counts (50, 150, 300, and 500). Values represent the posterior model minus true value.

For density, in scenarios with a low presence of species (presence: 0.40), the simulations show that increasing the number of survey visits from one to three did not improve the negative bias or precision of density estimates (Fig. 3). For moderately occurring species (presence: 0.6), single visit surveys resulted in negatively biased estimates when the probability of availability was low (probability of availability: 0.4); however, this bias was significantly reduced by implementing three repeated visits, with an associated increase in precision with more point counts. Additionally, increasing the probability of availability from 0.4 to 0.8 improves the accuracy of density estimates for both single and triple visit surveys. Survey methods generally produced satisfactory bias in density estimates across most scenarios, suggesting robustness in the survey design for frequently occurring species irrespective of the probability of availability (species with a high presence (presence: 0.8)).

### Bird surveys

**Fig. 4:**
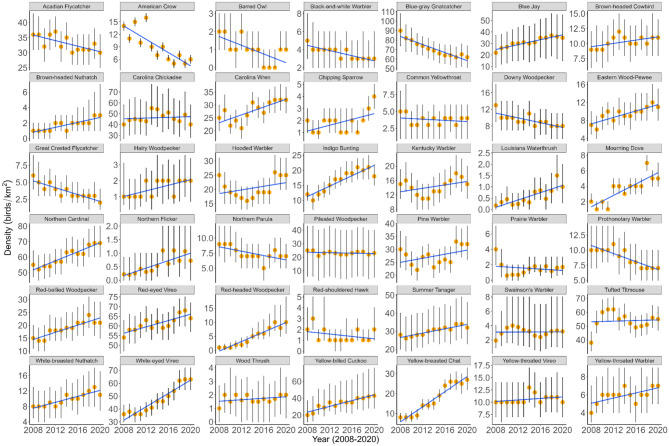
Density estimates (birds/km^2^) for 42 bird species predicted from a regional hybrid, time-to-detection hierarchical Bayesian model accounting for temporal variability at three National Wildlife refuges in Oklahoma and Texas from 2008–2020. For each bird species, solid lines represent the mean density predictions, while the error bars denote the 95% credibility intervals on these posterior distributions. The closed circles correspond to annual, species-specific density estimates. Each panel corresponds to a different species. The scientific names are reported in Table 2. Note: Not all trends plotted are significantly different from a zero slope. Significant trends are described in the “Results” section.

From 2008 to 2020, we conducted avian surveys at points located on the lands of three NWRs (Table 2). We detected 12,561 individual birds and 85 species, of which 57 species were detected at two refuges or more and 39 detected at all three. Forty-two species were observed at more than 10% of the point counts across all three refuges. Acadian Flycatcher (*Empidonax virescens*; 33 birds/km^2^; 95% credibility intervals (CI) 30–37), Carolina Wren (*Thryothorus ludovicianus*; 28 birds/km^2^; 95% CI 25–31), Blue-Gray Gnatcatcher (*Polioptila caerulea*; 77 birds/km^2^; 95% CI 67–88), Northern Cardinal (*Cardinalis cardinalis*; 61 birds/km^2^; 95% CI 54–68), Pine Warbler (*Setophaga pinus*; 27 birds/km^2^; 95% CI 24–31), Red-eyed Vireo (*Vireo olivaceus*; 61 birds/km^2^; 95% CI 57–66), Tufted Titmouse (*Baeolophus bicolor*; 49 birds/km^2^; 95% CI 44–54), White-eyed Vireo (*Vireo griseus*; 47 birds/km^2^; 95% CI 42–52), and Yellow-billed Cuckoo (*Coccyzus americanus*; 34 birds/km^2^; 95% CI 23–58) had the greatest estimates of densities and probability of availability (Fig. 4; Table 2). Focal species of interest, including the Acadian Flycatcher referenced above, Brown-headed Nuthatch (*Sitta pusilla*; 2 birds/km^2^; 95% CI 1–3), Kentucky Warbler (13 birds/km^2^; 95% CI 11–16), Louisiana Waterthrush (*Parkesia motacilla*; 1 birds/km^2^; 95% CI 0–2), Northern Parula (*Setophaga americana*; 7 birds/km^2^; 95% CI 6–10), Prairie Warbler (*Setophaga discolor*; 2 birds/km^2^; 95% CI 0–3), Prothonotary Warbler (*Protonotaria citrea*; 9 birds/km^2^; 95% CI 7–11), Red-headed Woodpecker (*Melanerpes erythrocephalus*; 19 birds/km^2^; 95% CI 15–24), Swainson’s Warbler (3 birds/km^2^; 95% CI 0–8), and Yellow-throated Warbler (*Setophaga dominica*; 6 birds/km^2^; 95% CI 5–8), had lower densities (Fig. 4), probability of availability, and probability of detection estimates than most species we observed (Appendices).

**Table 2:** Estimated avian density and 95% credibility intervals (CI) for avian species in bottomland hardwood and riparian forest at Little River National Wildlife Refuge, Oklahoma and Caddo Lake and Little Sandy National Wildlife Refuges, Texas that were surveyed from 2008 to 2020.

Species		Availability		Detection		AR(1)		AR(2)		Density (birds/km^2^)	
Common and scientific name	$n$	${p}_{a}$	95% CI	${p}_{d}$	95% CI	${\gamma }_{1}$	95% CI	${\gamma }_{2}$	95% CI	D	95% CI
Acadian Flycatcher (ACFL), *Empidonax virescens*	668	0.92	0.89–0.95	0.27	0.25–0.29	0.97	0.94–0.99	–	–	33	30–37
American Crow (AMCR), *Corvus brachyrhynchos*	474	0.87	0.81–0.91	0.95	0.93–0.97	0.92	0.88–0.96	0.08	0.01–0.14	9	7–10
Barred Owl (BADO), *Strix varia*	48	0.78	0.36–0.94	0.96	0.93–0.99	0.93	0.82–0.99	–	–	1	0–2
Black-and-white Warbler (BAWW), *Mniotilta varia*	42	0.88	0.87–0.92	0.25	0.18–0.31	0.95	0.91–0.99	–	–	4	2–5
Blue-gray Gnatcatcher (BGGN), *Polioptila caerulea*	429	0.81	0.74–0.87	0.10	0.08–0.12	0.59	0.35–0.81	0.40	0.19–0.65	77	67–88
Brown-headed Cowbird (BHCO), *Molothrus ater*	182	0.37	0.21–0.52	0.32	0.28–0.38	0.94	0.90–0.98	–	–	10	8–12
Brown-headed Nuthatch (BHNU), *Sitta pusilla*	26	0.79	0.68–0.91	0.35	0.18–0.56	0.64	0.41–0.89	–	–	2	1–3
Blue Jay (BLJA), *Cyanocitta cristata*	235	0.32	0.12–0.55	0.55	0.48–0.63	0.92	0.88–0.97	–	–	31	16–49
Carolina Chickadee (CACH), *Poecile carolinensis*	314	0.54	0.40–0.67	0.23	0.21–0.26	0.89	0.84–0.94	–	–	46	32–66
Carolina Wren (CARW), *Thryothorus ludovicianus*	738	0.84	0.79–0.88	0.46	0.42–0.50	0.47	0.31–0.66	0.44	0.25–0.60	28	25–31
Chipping Sparrow (CHSP), *Spizella passerina*	45	0.41	0.08–0.78	0.56	0.52–0.56	0.94	0.88–0.99	–	–	2	1–3
Common Yellowthroat (COYE), *Geothlypis trichas*	63	0.73	0.12–0.92	0.33	0.25–0.42	0.74	0.25–0.99	0.23	0.01–0.69	4	2–10
Downy Woodpecker (DOWO), *Picoides pubescens*	156	0.66	0.61–0.76	0.33	0.28–0.40	0.93	0.84–0.99	–	–	9	7–11
Eastern Wood-pewee (EAWP), *Contopus virens*	235	0.70	0.55–0.81	0.57	0.49–0.66	0.98	0.93–0.99	–	–	10	7–13
Great Crested Flycatcher (GCFL), *Myiarchus crinitus*	92	0.64	0.35–0.85	0.34	0.27–0.43	0.13	0.02–0.42	0.87	0.59–0.99	4	3–5
Hairy Woodpecker (HAWO), *Picoides villosus*	31	0.69	0.61–0.84	0.46	0.27–0.56	0.55	0.05–0.99	–	–	2	1–3
Hooded Warbler (HOWA), *Setophaga citrina*	318	0.80	0.72–0.87	0.34	0.31–0.38	0.30	0.13–0.52	0.56	0.37–0.72	20	18–24
Indigo Bunting (INBU), *Passerina cyanea*	472	0.89	0.85–0.92	0.47	0.43–0.52	0.96	0.93–0.99	–	–	17	15–19
Kentucky Warbler (KEWA), *Geothlypis formosa*	255	0.72	0.60–0.82	0.39	0.34–0.44	0.30	0.12–0.54	0.46	0.27–0.61	13	11–16
Louisiana Waterthrush (LOWA), *Parkesia motacilla*	15	0.74	0.62–0.93	0.55	0.46–0.56	0.94	0.79–0.99	–	–	1	0–2
Mourning Dove (MODO), *Zenaida macroura*	130	0.78	0.65–0.87	0.96	0.94–0.98	0.96	0.90–0.99	–	–	4	3–4
Northern Cardinal (NOCA), *Cardinalis cardinalis*	1066	0.72	0.66–0.77	0.38	0.36–0.41	0.89	0.86–0.92	–	–	61	54–68
Northern Flicker (NOFL), *Colaptes auratus*	9	0.73	0.61–0.92	0.50	0.24–0.99	0.92	0.43–0.99	–	–	1	0–2
Northern Parula (NOPA), *Setophaga americana*	201	0.87	0.79–0.93	0.37	0.32–0.43	0.96	0.94–0.98	–	–	7	6–10
Pileated Woodpecker (PIWO), *Dryocopus pileatus*	263	0.31	0.08–0.57	0.95	0.92–0.98	0.92	0.89–0.95	–	–	16	6–46
Pine Warbler (PIWA), *Setophaga pinus*	423	0.82	0.77–0.87	0.36	0.33–0.39	0.56	0.35–0.84	0.34	0.08–0.55	27	24–31
Prairie Warbler (PRAW), *Setophaga discolor*	25	0.87	0.67–0.97	0.40	0.27–0.59	0.74	0.40–0.99	0.27	0.01–0.51	2	0–3
Prothonotary Warbler (PROW), *Protonotaria citrea*	232	0.77	0.64–0.88	0.38	0.33–0.44	0.98	0.97–0.99	–	–	9	7–11
Red-bellied Woodpecker (RBWO), *Melanerpes carolinus*	458	0.62	0.50–0.73	0.60	0.53–0.67	0.92	0.88–0.95	–	–	19	15–24
Red-eyed Vireo (REVI), *Vireo olivaceus*	1175	0.90	0.88–0.92	0.29	0.27–0.31	0.92	0.90–0.94	–	–	61	57–66
Red-headed Woodpecker (RHWO), *Melanerpes erythrocephalus*	93	0.67	0.39–0.84	0.55	0.43–0.69	0.26	0.01–0.83	0.67	0.11–0.96	5	3–8
Red-shouldered Hawk (RSHA), *Buteo lineatus*	67	0.69	0.32–0.89	0.94	0.91–0.97	0.91	0.86–0.97	–	–	2	1–4
Summer Tanager (SUTA), *Piranga rubra*	375	0.54	0.39–0.68	0.35	0.31–0.38	0.90	0.86–0.95	–	–	30	23–42
Swainson’s Warbler (SWWA), *Limnothlypis swainsonii*	36	0.63	0.46–0.85	0.55	0.46–0.56	0.96	0.87–0.99	–	–	3	0–8
Tufted Titmouse (TUTI), *Baeolophus bicolor*	914	0.79	0.74–0.84	0.33	0.30–0.35	0.62	0.12–0.91	0.28	0.01–0.78	49	44–54
White-breasted Nuthatch (WBNU), *Sitta carolinensis*	112	0.54	0.46–0.70	0.31	0.26–0.38	0.94	0.86–0.99	–	–	10	7–13
White-eyed Vireo (WEVI), *Vireo griseus*	798	0.86	0.82–0.89	0.28	0.26–0.30	0.88	0.85–0.91	–	–	47	42–52
Wood Thrush (WOTH), *Hylocichla mustelina*	14	0.19	0.03–0.53	0.88	0.46–0.95	0.91	0.84–0.99	–	–	4	1–16
Yellow-breasted Chat (YBCH), *Icteria virens*	395	0.85	0.79–0.90	0.47	0.42–0.52	0.95	0.93–0.98	–	–	17	15–19
Yellow-billed Cuckoo (YBCU), *Coccyzus americanus*	541	0.38	0.13–0.56	0.59	0.03–0.65	0.46	0.08–0.89	0.44	0.03–0.85	34	23–58
Yellow-throated Vireo (YTVI), *Vireo flavifrons*	197	0.72	0.55–0.84	0.31	0.26–0.35	0.92	0.86–0.98	–	–	11	8–15
Yellow-throated Warbler (YTWA), *Setophaga dominica*	199	0.79	0.65–0.88	0.49	0.41–0.59	0.94	0.91–0.97	–	–	6	5–8

The numbers of detections (*n*) during 10-min point counts were used to estimate species-specific probability of availability (${p}_{a}$), probability of detection (${p}_{d}$), and autoregressive model of order 1 and 2 parameters ($\gamma$).

Our analysis of bird detection data and the precision of density estimates revealed that the ability to characterize species trends over time varied among the refuges (Appendices). Our comprehensive “All Refuge” analysis, which combined data across refuges, improved the precision of density and trend estimates. We identified declining trends for the Blue-gray Gnatcatcher (− 6% year^−2^) and Great Crested Flycatcher (*Myiarchus crinitus*; − 7% year^-2^), alongside strongly supported increasing trends for species such as Carolina Wren (+ 5% year^-2^), Indigo Bunting (*Passerina cyanea*; + 4% year^−1^), Mourning Dove (*Zenaida macroura*; + 30% year^-1^), Red-headed Woodpecker (+ 56% year^−2^), White-eyed Vireo (+ 5% year^−1^), and Yellow-breasted Chat (*Icteria virens*; + 12% year^−1^; Fig. 4; Table 2). Many species exhibited sustained trends, including the Kentucky Warbler (+ 0.58% year^−2^), Northern Parula (− 0.87% year^−1^), Pine Warbler (+ 1.42% year^−2^), Prairie Warbler (*Setophaga discolor*; + 2.23% year^-2^), and Tufted Titmouse (+ 3.79% year^−2^; Fig. 4; Table 2). Other focal species like Common Yellowthroat (*Geothlypis trichas*; CV = 0.78), Louisiana Waterthrush (CV = 1.01), Northern Flicker (*Colaptes auratus*; CV = 0.85), and Swainson’s Warbler (CV = 0.56), lacked sufficient detections which led to imprecise density estimates, hindering our ability to discern trends over time (Fig. 4; Table 2).

The refuge-specific analyses revealed gaps in data sufficiency for modeling many species. For example, the “All Refuge” analyses contained 201 detections of Northern Parula (7 birds/km^2^; 95% CI 6–10) and 232 detections of Prothonotary Warbler (9 birds/km^2^; 95% CI 7–11). Yet, at Caddo Lake National Wildlife Refuge (NWR), we had only 20 detections for Northern Parula with trends indicating 1–2 birds and 10 detections for Prothonotary Warbler with trends of 0–3 birds, both with wide credibility intervals (Appendix). Both species exhibited wide credibility intervals, reflecting high uncertainty (Appendices). For other species, positive trends were identified, including Carolina Chickadee (*Poecile carolinensis*; 44 birds/km^2^; 95% CI 30–74), Carolina Wren (22 birds/km^2^; 95% CI 18–27), Eastern Wood-Pewee (*Contopus virens*; 5 birds/km^2^; 95% CI 5–7), Hooded Warbler (*Setophaga citrina*; 21 birds/km^2^; 95% CI 18–24), Indigo Bunting (18 birds/km^2^; 95% CI 16–21), Kentucky Warbler (13 birds/km^2^; 95% CI 8–21), Northern Cardinal (51 birds/km^2^; 95% CI 43–62), Red-eyed Vireo (39 birds/km^2^; 95% CI 35–44), White-eyed Vireo (36 birds/km^2^; 95% CI 30–41), and Yellow-breasted Chat (18 birds/km^2^; 95% CI 16–21). At Little River NWR, trends were either flat or negative for species such as Acadian Flycatcher (52 birds/km^2^; 95% CI 44–61), Blue-gray Gnatcatcher (48 birds/km^2^; 95% CI 38–62), Carolina Wren (21 birds/km^2^; 95% CI 18–26), Eastern Wood-Pewee (7 birds/km^2^; 95% CI 5–8), Hooded Warbler (8 birds/km^2^; 95% CI 6–12), Indigo Bunting (7 birds/km^2^; 95% CI 5–10), Kentucky Warbler (10 birds/km^2^; 95% CI 8–12), and Yellow-breasted Chat (7 birds/km^2^; 95% CI 5–9), with no strongly supported positive trends detected (Appendices). At Little Sandy NWR, our data allowed for precise trend characterization for species including Blue-gray Gnatcatcher (46 birds/km^2^; 95% CI 44–49), Carolina Wren (37 birds/km^2^; 95% CI 35–45), Indigo Bunting (20 birds/km^2^; 95% CI 17–23), Pileated Woodpecker (*Dryocopus pileatus*; 17 birds/km^2^; 95% CI 13–20), Pine Warbler (10 birds/km^2^; 95% CI 7–13), and Yellow-billed Cuckoo (26 birds/km^2^; 95% CI 22–30), all lacking directional trends (Appendices). However, the considerable uncertainty for other species with few detections—regardless of refuge—underscores the challenge of insufficient data in accurately estimating true densities and detecting population trends (Appendices).

### Number of bird point counts

**Fig. 5:**
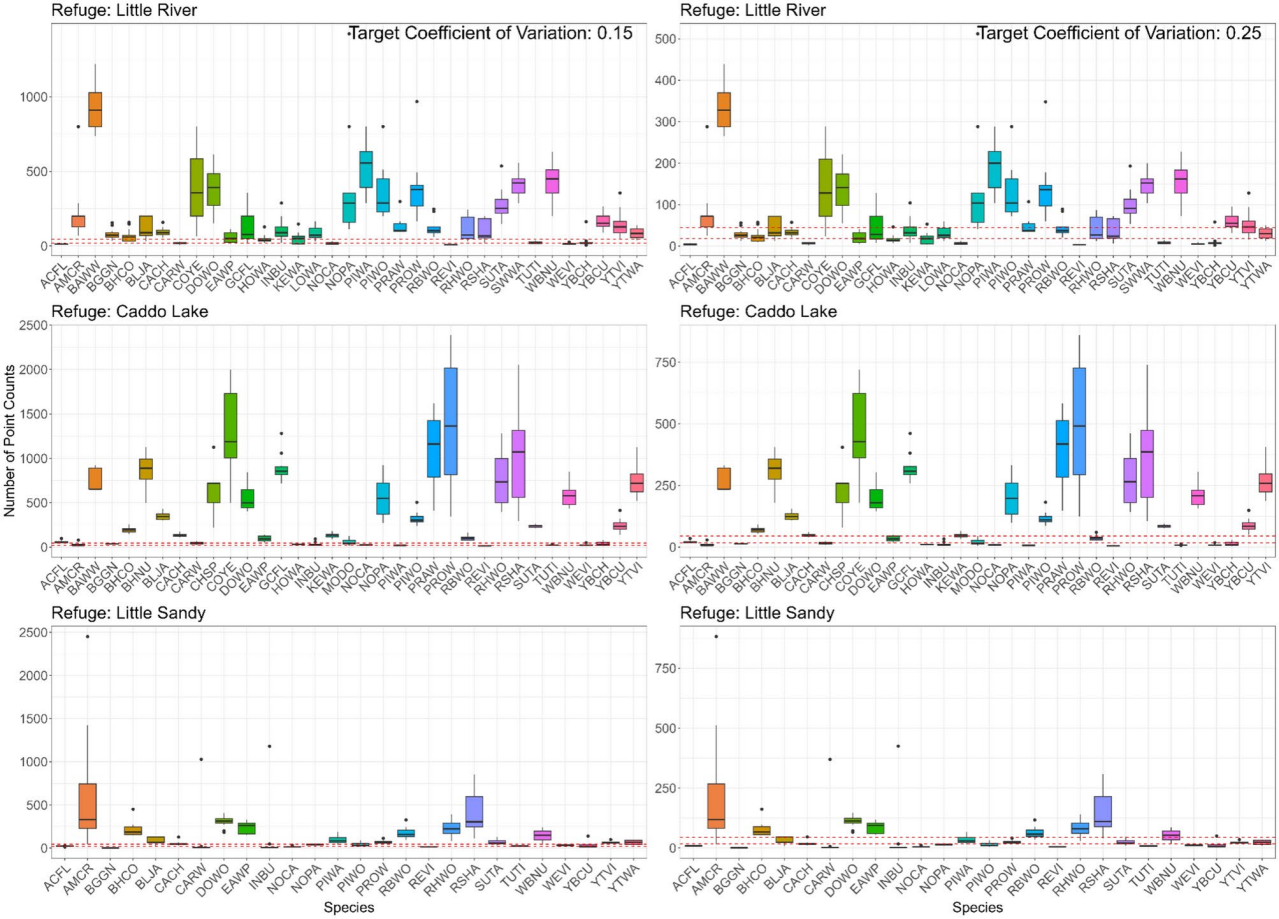
Boxplots displaying estimates of species-specific point counts for Little River, Caddo Lake, and Little Sandy National Wildlife Refuges, comparing two target coefficients of variation (0.15 and 0.25). The dashed lines (red) represent the current survey levels at each refuge, offering a benchmark for evaluating the variability in the necessary point counts against conservation objectives. The different colors of the boxplots correlate to individual species. The species acronyms, along with their common and scientific names, are reported in Table 2.

Our analysis estimated the requisite number of bird point counts per species, annually, and across each refuge, to achieve coefficient of variation (CV) targets of 0.15 and 0.25 (Table 3). For a CV of 0.15, only the most abundant species could meet survey requirements with the existing number of bird point counts at each refuge. The range of required point counts for less abundant, rarely occurring species varied significantly by refuge: Black-and-white Warbler (*Mniotilta varia*; 500–1200), Common Yellowthroat (500–1800), Kentucky Warbler (30–1000), Louisiana Waterthrush (45–500), Northern Flicker (≥ 500), Northern Parula (100–750), Pine Warbler (40–600), Prothonotary Warbler (25–100), Summer Tanager (*Piranga rubra*; 100–300), Swainson’s Warbler (400–1700), Yellow-billed Cuckoo (100–300), Yellow-throated Vireo (*Vireo flavifrons*; 25–1000), and Yellow-throated Warbler (40–100) (Fig. 5). Setting a target CV of 0.25 reduced the number of bird point counts needed to achieve precise density estimates. For instance, Black-and-white Warbler required 200 to 400 point counts, Common Yellowthroat needed 50–600, Kentucky Warbler ranged from 20 to 300, Louisiana Waterthrush from 25 to 450, Northern Flicker ≥ 200, Northern Parula between 90 and 260, Pine Warbler from 25 to 250, Prothonotary Warbler ranged 50–500, Summer Tanager from 25 to 200, Swainson’s Warbler between 180 and 550, Yellow-billed Cuckoo needed 50–100, Yellow-throated Vireo required 50–260, and Yellow-throated Warbler ranged from 20 to 100 (Fig. 5).

**Table 3:** Required sample size (number of bird point counts) for achieving target coefficients of variation (CV) of 0.15 and 0.25 in density estimates of selected bottomland and upland bird species at Caddo Lake, Little River, and Little Sandy National Wildlife Refuges.

Refuge	Species	Target CV: 0.15			Target CV: 0.25		
		Mean	SD	Range	Mean	SD	Range
Caddo Lake	Acadian Flycatcher *Emidonax virescens*	60	15	45–97	21	5	16–35
	Brown-headed Nuthatch *Sitta pusilla*	863	195	500–1125	311	70	180–405
	Kentucky Warbler *Geothlypis formosa*	133	26	92–180	48	9	33–65
	Louisiana Waterthrush *Parkesia motacilla*	–	–	–	–	–	–
	Prairie Warbler *Setophaga discolor*	1089	448	411–1620	392	161	148–583
	Prothonotary Warbler *Protonotaria citrea*	1388	812	344–2386	500	292	124–859
	Red-headed Woodpecker *Melanerpes carolinus*	782	317	395–1280	281	114	142–461
	Swainson’s Warbler *Limnothlypis swainsonii*	1257	276	980–1531	453	99	353–551
	Wood Thrush *Hylocichla mustelina*	319	163	107–526	115	59	38–189
	Yellow-throated Warbler *Setophaga dominica*	86	67	45–263	31	24	16–95
Little River	Acadian Flycatcher *Emidonax virescens*	13	4	7–20	5	2	3–7
	Brown-headed Nuthatch *Sitta pusilla*	–	–	–	–	–	–
	Kentucky Warbler *Geothlypis formosa*	51	39	10–147	19	14	4–53
	Louisiana Waterthrush *Parkesia motacilla*	89	53	32–166	32	19	12–60
	Prairie Warbler *Setophaga discolor*	144	80	94–298	52	29	34–107
	Prothonotary Warbler *Protonotaria citrea*	386	204	165–968	139	73	60–348
	Red-headed Woodpecker *Melanerpes carolinus*	119	91	32–242	43	32	12–87
	Swainson’s Warbler *Limnothlypis swainsonii*	405	84	288–556	146	30	104–200
	Wood Thrush *Hylocichla mustelina*	670	197	408–886	241	71	147–319
	Yellow-throated Warbler *Setophaga dominica*	85	34	37–139	30	12	13–50
Little Sandy	Acadian Flycatcher *Emidonax virescens*	23	5	12–31	9	1	7–11
	Brown-headed Nuthatch *Sitta pusilla*	344	216	216–593	124	78	78–214
	Kentucky Warbler *Geothlypis formosa*	848	284	481–1152	305	102	173–415
	Louisiana Waterthrush *Parkesia motacilla*	–	–	–	–	–	–
	Prairie Warbler *Setophaga discolor*	–	–	–	–	–	–
	Prothonotary Warbler *Protonotaria citrea*	71	20	46–113	25	7	17–41
	Red-headed Woodpecker *Melanerpes carolinus*	231	109	83–392	83	39	30–141
	Swainson’s Warbler *Limnothlypis swainsonii*	–	–	–	–	–	–
	Wood Thrush *Hylocichla mustelina*	–	–	–	–	–	–
	Yellow-throated Warbler *Setophaga dominica*	65	24	31–95	23	9	11–34

The species were selected based on their ecological relevance to bottomland hardwood and riparian forests, detectability during surveys, and importance for management objectives.

**Fig. 6:**
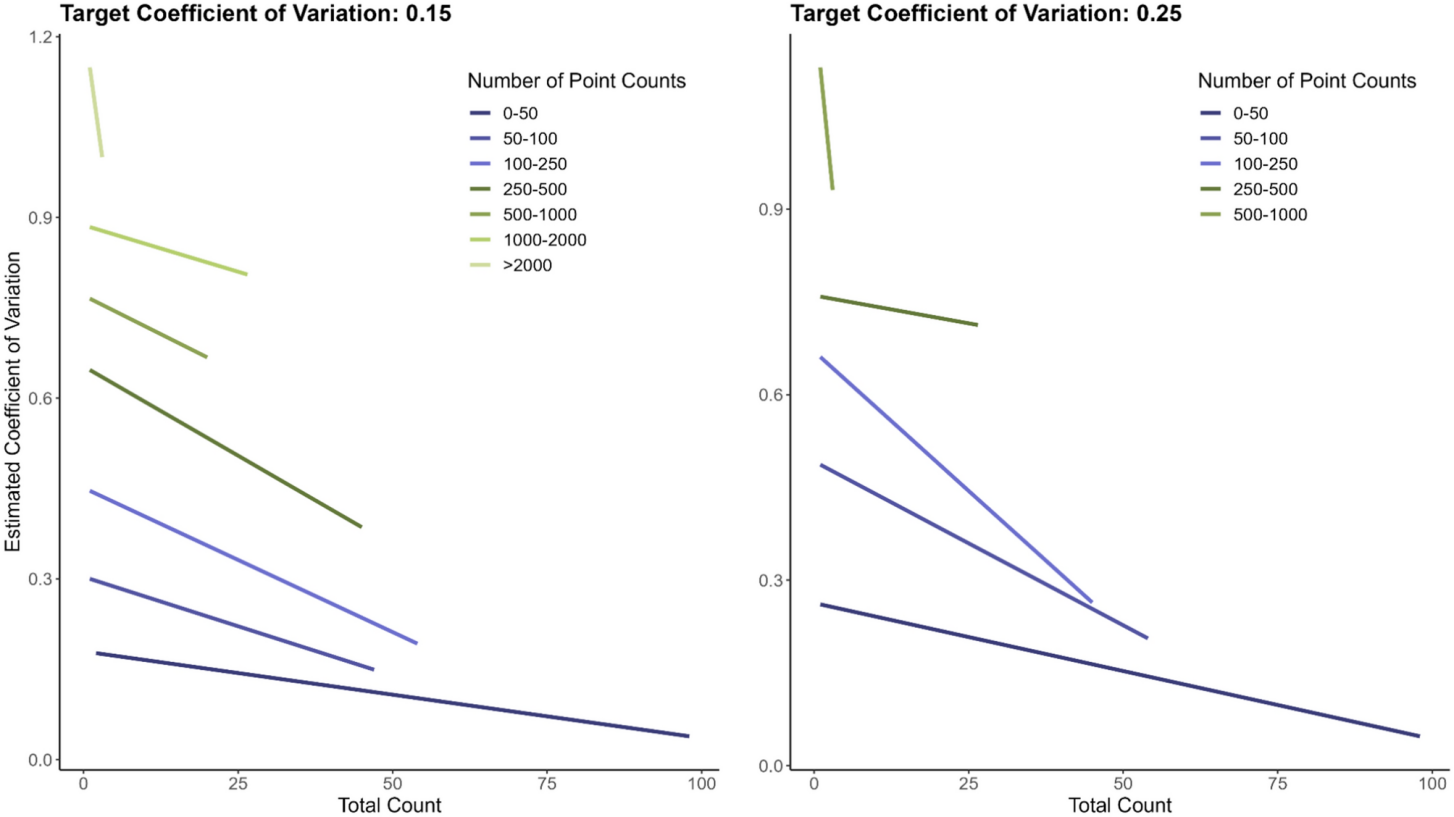
The estimated relationship between the total count of bird observations (x-axis) and the estimated coefficient of variation (CV) (y-axis) for achieving target CVs of 0.15 and 0.25, across different numbers of point counts (z-axis) binned from 0 to 50, 50 to 100, 100 to 250, 500 to 1000, 1000 to 2000, and > 2000.

We integrated results from our power analyses and field efforts to generate an XYZ figure for informing future and related avian studies (Fig. 6). The number of point counts required to achieve a desired CV decreased as the CV produced from a pilot study increased and the number of required points rose as the total counts of individual bird observations declined (Fig. 6). The reduction in number of point counts was more pronounced with a CV of 0.25 compared to 0.15. Typically, for a study resulting in a total count of 50 individuals of a single species and a CV ~ 0.30, approximately 100–250 point counts would be necessary to achieve a target CV of 0.15, while < 100 point counts should meet a CV of 0.25 (Fig. 6). When the estimated CV exceeded 0.30 with 50 observed individuals, ≥ 250 point counts were required to reach a CV of 0.15, and over 100 to achieve a CV of 0.25. For a CV > 0.60 acquired from a study, with fewer than 50 individuals were observed, the requisite point counts increased to over 500 to obtain a target of 0.15 and ~ 250 for a target of 0.25.

Our literature search revealed the minimum sample size required to estimate density from multispecies surveys was 241 point counts on average, ranging from 20 to 828 (Table 4). For individual species, the Acadian Flycatcher and the American Crow (*Corvus brachyrhynchos*) had average minimum sample sizes of 94 and 264. The Downy Woodpecker (*Picoides pubescens*) and the Great Crested Flycatcher had the highest average minimum sample sizes of 920 and 849. The Hooded Warbler and Kentucky Warbler had an average minimum sample size of 314 (9–1655) and 286 (9–1561). The Northern Parula had an average minimum sample size of 130 (23–200), and Summer Tanagers and Yellow-throated Vireos both had comparable average minimum sample sizes of 113 and 124.

**Table 4:** The average minimum sample size requirements for bottomland hardwood and riparian avian species surveys, as reported from 23 research studies.

Species	Average minimum sample size	Range	References
Multispecies survey (general)	241	20–828	Augenfeld et al.^23^, Blake^24^, Bub et al.^25^, Buskirk and McDonald^26^, DeSante^27^, Hanowski and Niemi^28^, Howe et al.^29^, Heckscher^30^, Heltzel and LeBerg^31^, Hutto et al.^32^, Latta et al.^33^, LeGrand^34^, Pierce and King^35^, Preston et al.^36^, Rolek et al.^37^, Rotenberry and Knick^38^, Smith et al.^39^, Swanson et al.^40^, Thompson and Schwalbach^41^, Thompson et al.^42^, Twedt and Somershoe^43^
Acadian Flycatcher *Empidonax virescens*	94	23–287	Smith et al.^39^,Thompson et al.^44^
American Crow *Corvus brachyrhynchos*	264	181–347	Thompson et al.^44^
American Redstart *Setophaga ruticilla*	124	9–200	Smith et al.^39^
Blue-gray Gnatcatcher *Polioptila caerulea*	108	44–200	Smith et al.^39^
Brown-headed Cowbird *Molothrus ater*	178	44–467	Smith et al.^39^,Thompson et al.^44^
Carolina Chickadee *Poecile carolinensis*	200	–	Smith et al.^39^
Carolina Wren *Thryothorus ludovicianus*	176	33–994	Smith et al.^39^
Downy Woodpecker *Picoides pubescens*	920	632–1208	Thompson et al.^44^
Eastern Wood-Pewee *Contopus virens*	306	210–401	Thompson et al.^44^
Great Crested Flycatcher *Myiarchus crinitus*	849	583–1114	Thompson et al.^44^
Hooded Warbler *Setophaga citrina*	314	9–1655	Smith et al.^39^, Thompson and Schwalbach^41^
Indigo Bunting *Passerina cyanea*	205	9–764	Smith et al.^39^, Thompson and Schwalbach^41^
Kentucky Warbler *Geothlypis formosa*	286	9–1561	Smith et al.^39^, Thompson and Schwalbach^41^
Northern Cardinal *Cardinalis cardinalis*	99	20–200	Smith et al.^39^,^45^
Northern Parula *Setophaga americana*	130	23–200	Smith et al.^39^
Ovenbird *Seiurus aurocapilla*	226	155–297	Thompson et al.^44^
Pileated Woodpecker *Dryocopus pileatus*	279	189–368	Thompson et al.^44^
Prothonotary Warbler *Protonotaia citrea*	93	23–200	Smith et al.^39^,^45^
Red-bellied Woodpecker *Melanerpes carolinus*	115	33–498	Smith et al.^39^, Thompson and Schwalbach^41^
Red-eyed Vireo *Vireo olivaceus*	130	23–398	Smith et al.^39^,^45^, Thompson et al.^44^
Rufous-sided Towhee *Pipilo erythropthalmus*	365	9–2066	Smith et al.^39^, Thompson and Schwalbach^41^
Scarlet Tanager *Piranga olivacea*	207	142–272	Thompson and Schwalbach^41^
Summer Tanager *Piranga rubra*	113	9–200	Smith et al.^39^
Swainson’s Warbler *Limnothlypis swainsonii*	61	–	Peters et al.^46^
Tufted Titmouse *Baeolophus bicolor*	84	37–200	Smith et al.^39^, Thompson and Schwalbach^41^
White-breasted Nuthatch *Sitta carolinensis*	636	437–835	Thompson and Schwalbach^41^
Wood Thrush *Hylocichla mustelina*	135	15–414	Smith et al.^39^,^45^, Thompson and Schwalbach^41^
Worm-eating Warbler *Helmitheros vermivorum*	417	286–547	Thompson and Schwalbach^41^
Yellow-billed Cuckoo *Coccyzus americanus*	126	37–419	Smith et al.^39^, Thompson and Schwalbach^41^
Yellow-throated Vireo *Vireo flavifrons*	124	9–200	Smith et al.^39^

A ‘multispecies survey’ or ‘general’ refers to surveys in which detections of multiple avian species were recorded.

## Discussion

We analyzed data from point counts to evaluate the effectiveness of current survey methods and estimate the density of bottomland hardwood and riparian forest birds at three national wildlife refuges in northeastern Texas and southern Oklahoma, USA. This work aims to assess the applicability of the existing survey approach, inform survey revision, and advise future, similar survey efforts in forested ecosystems. Accurate and precise estimates of species abundance and density are essential to forest managers to implement silvicultural treatments that support bottomland hardwood and riparian forest bird species. Our findings indicate that the number of bird point counts conducted annually, combined with the resulting species detections, was insufficient to clarify trends in bird density—particularly for rare species prioritized in management objectives, where fewer than 50 detections were recorded over the study period spanning 2008–2020. Our results underscore the need for a refined survey approach that prioritizes generating sufficient detections of target species to improve the precision of density and trend estimates, ensuring that management efforts are effectively aligned with conservation goals.

Our analysis highlights the challenge of collecting sufficient data on species of management concern using standard multispecies survey designs, particularly when survey site allocation lacks careful evaluation. The current survey was implemented without consideration of clearly defined objectives, precision goals, or the use of a formal process in site allocation. To address these limitations, a randomized placement of survey points, stratified by habitat and management zones, may be necessary to accurately estimate bird density and distribution. Additionally, strategies like repeat site visits within a year, spatially-adaptive designs, or two-phase sampling could enhance detections during annual surveys^47,48^. Without these refinements, surveys risk over-representing common species while failing to capture adequate data on rarer species with higher conservation priority. Clearly defining objectives, incorporating biological hypotheses, and using pilot survey data to guide design and resource allocation can improve survey effectiveness and better support management goals^17,49,50^.

Our simulation results demonstrated that repeated visits to point count locations within a survey year significantly enhanced the precision and accuracy of avian population estimates, particularly for species with moderate to high presence levels. Repeated surveys reduced the bias observed in single-visit surveys when estimating probabilities of availability and density^42,51^. However, for species with low presence—those often prioritized by wildlife managers—the variance in estimates remained high, and density estimates were negatively biased, regardless of whether a site was visited once or multiple times within a year, a function of low detection rates or lack of data going into the model. Increasing the total number of unique survey points improved precision but did not reduce bias for these low-presence species. These findings suggest that while repeated visits can improve survey outcomes, their effectiveness depends heavily on a well-considered survey design. Simply increasing the number of point counts or revisiting the same locations may not yield substantial benefits unless the distribution and habitats that support rare or less detectable species are considered, especially habitat specialists. By focusing efforts in areas where these species are likely to occur and incorporating habitat-specific features, approaches such as two-stage sampling or stratified random sampling^47,48^ can significantly increase detection rates. This approach reduces uncertainty, improves the ability to estimate density and trends for focal species, and helps managers meet precision goals^17,42^.

To achieve the necessary precision for management-relevant species, survey approaches must align with well-defined objectives that are specific, measurable, achievable, and time-defined^17^. In this case, the lack of clarity in survey objectives impaired the design and effectiveness of the effort. While the survey aimed to assess changes in bird populations and link them to habitat management, limited data from a small number of sites often left population trends unclear. Additionally, habitat variables were either not measured at an appropriate scale or were inconsistently recorded throughout the survey, further complicating efforts to connect population trends to management actions and forest inventory measurements. Despite these challenges, the survey effectively tracked density changes over time for some abundance and readily observed species at individual refuges. For rare or management-priority species, aggregating data across refuges and years improved the precision of density estimates, allowing trend estimation for more species. However, this aggregation obscured local ecological variations and dynamics at individual refuges, which are critical for informing refuge-specific management and silvicultural practices^52^.

Most multispecies bottomland hardwood and riparian forest bird surveys reported an average of 241 bird point counts necessary to cover larger conservation areas (Table 4). While the refuges we monitored covered a smaller areas, making 241 point counts potentially excessive given space, these areas would still benefit from increased sample sizes and reallocating point counts using a randomized design based on specific habitat conditions. Increasing the number of point counts is significant because it represents the survey effort needed to achieve adequate precision (e.g., CV of 0.25 or lower) for estimating bird densities and detecting trends across multiple species (Table 4). Well-defined precision goals ensures sufficient data collection to reduce bias and improve the reliability of estimates, particularly for rare or less detectable species often prioritized in conservation efforts. For example, at Little River NWR, our analysis indicates that achieving moderate precision (CV_0.25_) for species like Louisiana Waterthrush and Swainson’s Warbler requires between 32 and 146 point counts (Table 3). Higher precision (CV_0.15_) demands significantly more effort, ranging from 89 to 405 point counts. At Caddo Lake NWR, achieving CV_0.15_ for Swainson’s Warbler necessitates a mean of 1257 point counts (Table 3). In contrast, Smith et al.^39^ reported that species like the Northern Parula and Summer Tanager require approximately 130 and 113 point counts, respectively (Table 4). However, our analysis estimated that achieving CV_0.25_ for these species would require at least 139 and 71 point counts. These estimates, along with others reported in Table 3, align with findings from previous studies, as reported in Table 4. They underscore the importance of conducting pilot studies to inform future survey designs that meet precision goals, as current efforts in our example fall short of necessary thresholds. Additionally, these findings highlight how survey effort requirements vary significantly by study area, year, and species. Addressing this variability and increasing survey effort where needed will improve data quality^53^, and better support decision-making, ultimately contributing to effective, data-driven management of bird populations in bottomland hardwood and riparian forests.

While using point count density (e.g., number of point counts per area) as a recommendation may initially seem practical, it does not fully account for habitat heterogeneity, which significantly influences detectability and abundance estimates. Habitat quality, variability, and species distributions are critical factors that determine the number of detections and the survey effort needed to meet precision goals. For highly detectable and abundant species, the current effort may align with the lower end of the recommended range, providing sufficient coverage. However, for less detectable or rare species, the current survey effort often falls short, particularly when higher precision levels are required for species-specific management objectives. Quantifying these gaps allows managers to assess whether their existing survey designs are adequate or require modification to meet conservation goals. Additionally, this evaluation provides a benchmark for other studies, helping to refine point count recommendations and improve the efficiency and reliability of future monitoring efforts. By addressing habitat heterogeneity and tailoring survey designs to species-specific needs, monitoring programs can better balance effort and precision, leading to improved data quality and more effective conservation outcomes.

Given the finite resources available for monitoring, prioritizing species during point counts is essential to maximize the cost-effectiveness and impact of surveys. Focusing on species that respond to silvicultural treatments allows surveys to provide actionable insights directly relevant to management goals. In our case, these priority species include the Acadian Flycatcher, Kentucky Warbler, Louisiana Waterthrush, Prothonotary Warbler, Yellow-throated Warbler, Swainson’s Warbler, Wood Thrush (*Hylocichla mustelina*), Brown-headed Nuthatch, Pine Warbler, Prairie Warbler, and Red-headed Woodpecker^54^. Prioritizing these species enables surveys to be strategically designed with optimized placement of survey points and increased frequency of visits to target habitats, improving data quality and detection rates. This tailored approach enhances the reliability of population estimates and trend analyses, ultimately providing more effective conservation assessments^43,55–57^. By aligning survey efforts with species-specific needs, limited resources can be used efficiently to support both species-specific management objectives and broader ecosystem restoration goals.

The density of bottomland hardwood and riparian forest bird species at a given location is influenced by external factors such as regional climatic patterns, neighboring land uses, and forest conditions on their wintering grounds, making it difficult to link even unbiased and precise estimates of bird density and density changes over time to specific forest conditions at a breeding site. A promising alternative is to identify target forest conditions favorable to these bird species and focus monitoring efforts on assessing whether such conditions are being achieved (Table 1). This approach allows managers to prioritize habitat restoration and monitoring under the assumption that birds will utilize the preferred habitats created. In its current form, the faunal survey generates insufficient and uninformative data on rare species of management concern, leading to inefficiencies and wasted resources. If faunal surveys remain a priority, they must be adequately designed and resourced to ensure they provide meaningful insights that support conservation and management objectives. By integrating strategic planning, resource allocation, and habitat-focused monitoring, managers can maximize the value of collected data and better align survey efforts with ecological and management goals.

Our research highlights the benefits of refined survey strategies that prioritize focal species and incorporate optimal design frameworks to improve data quality, particularly for rare and elusive species^48,58,59^. By strategically allocating point counts to focal areas within each refuge, the required sampling effort can be reduced compared to a multispecies approach. Future surveys can build upon the advanced statistical models used in this study, leveraging technologies such as airborne laser altimetry (i.e., discrete light detection and ranging (LiDAR)) to develop large-scale, multidate, and multispectral imagery for habitat characterization and high-resolution digital data layers of predicted target species densities^60^. Habitat-based approaches offer complementary or alternative strategies for assessing the effectiveness of silvicultural practices. The population monitoring methods developed in this study provide a foundation for USFWS-wide efforts to implement strategic, data-driven conservation monitoring aimed at evaluating species status and trends.

## Methods

### The model

We use a hierarchical Bayesian approach to fit alternative models that merge distance sampling and time removal techniques, aiming to estimate detection probability during avian point counts. These models provide an intuitive means of integrating alternative analytical structures. This includes the use of latent variables for modeling, data augmentation, and making inferences about shape and scale parameters, which in turn expresses the uncertainty in the posterior probability distribution of the model parameters^61^. Hierarchical Bayesian models are versatile and applicable to a wide range of capture-recapture experiments. This applies to unmarked and marked methods, like distance sampling that estimates abundance from unmarked individuals based on the distance of the individual from the observer. Hierarchical Bayesian models also allow for the straight forward development of models that account for the distinct processes that underlie avian surveys: abundance (denoted as ${N}_{{super}_{k}}$, which signifies the superpopulation of individuals in the surveyed region during the assessment period), perceptibility (${p}_{d}$)and availability (${p}_{a}$)^19,62,63^.

We define the observed elements of our model as individual counts, represented by $y$. These are sourced from either single visit or multi-visit point-count data, collected across points $k=1, 2,\dots .,K$ and on sampling occasions $t=1, 2,\dots .,T$ within a defined demographic closure period within a single year. The distances are classified into discrete classes $b=1, 2,\dots .,B$, reaching a specified maximum distance. Every detected bird has its time-to-detection recorded to a designated time interval $j=1, 2,\dots .,J$. Therefore, the observed data, represented as ${y}_{kt}$, can be organized into the matrix $Y=\left\{{y}_{kt}:k=1, 2,\dots .,K;t=1, 2,\dots .,T\right\}$. This matrix is perceived as a binomial outcome: ${y}_{kt}\sim Binomial\left({p}_{d},{N}_{{avail}_{kt}}\right)$, where ${p}_{d}$ signifies the consolidated probability of detection for point count $k$. To model the probability of perception, contingent on the observed data, the observation model was specified as ${dclass}_{i}\sim Categorical\left({\pi }_{d}^{c}\right)$ for individual $i$. The conditional multinomial cell probabilities for distance were formulated as ${\pi }_{{d}_{bk}}^{c}=\frac{{\pi }_{{d}_{bk}}}{\left({p}_{{d}_{k}}\right)}$ where ${\pi }_{{d}_{bk}}$ is the detection probability in distance class $b$ at point $k$ and ${p}_{{d}_{k}}$ represents the likelihood of detection in any distance category within the truncation radius at point $k$. Further, we defined the multinomial cell probability $\pi$ in distance class $b$ using a rectangular rule to approximate the integral. Here, that distance $r$ falls within the bounds of $b$ with width $\delta$ is ${\pi }_{rb}=Pr\left({r}_{b}-\frac{\delta }{2}\le r\le {r}_{b}+\frac{\delta }{2}\right)\sim {g\left(r\right)}_{bk}{f\left(r\right)}_{b}$. We assume that the detection probability diminishes with increasing distance from the observer, modeled by the half-normal detection function: $g{\left(r\right)}_{bk}=exp\left(-\frac{{{r}_{b}}^{2}}{2{\sigma }^{2}}\right)$, where $r$ denotes the radial distance and $\sigma$ is the scale parameter of the half-normal function. Meanwhile, $f{\left(r\right)}_{b}=\frac{2{r}_{b}{\delta }_{b}}{{B}_{d}^{2}}$ is the probability density function of radial distance from the observation point for each distance class, extending to the utmost truncation distance^64^. We derived the point-specific ${p}_{{d}_{k}}$ by aggregating the multinomial cell probability ${\pi }_{{d}_{bk}}$, which is the probability of an entity being detected at point $k$ in distance bin $b$, over all $b$ classes, as $\sum_{b=1}^{B}{\pi }_{{d}_{bk}}$.

Further, ${N}_{{avail}_{kt}}$ denotes individuals available for distance sampling during the survey period at point count $k$. It is assumed that ${N}_{kt}$ follows a binomial distribution: ${N}_{{avail}_{kt}}\sim Binomial\left(\phi , {N}_{{pres}_{kt}}\right)$. We begin again with the observation model, given ${y}_{kt}$. The second component of this is associated with the ${tinterval}_{i}\sim Categorical\left({\pi }_{a}^{c}\right)$ for individual $i$. The model factors in availability using the random temporary emigration model, wherein availability, is represented by $\phi$. In context of time-removal sampling, $\phi$ is associated with a per-period availability parameter ${p}_{a}$. Availability (${p}_{a}$) refers to the likelihood that a bird is present and available for detection during the survey period, which signifies the probability that an entity is present during any subsampling interval $j$. This concept is modeled using time-removal data, where the first detection times of birds are recorded in predefined time intervals (e.g., 0–3 min, 4–5 min, 6–10 min). These entities are modeled as Bernoulli random variables with conditional probabilities given by ${\pi }_{a}^{c}={p}_{a}/\phi$. The relationship between ${p}_{a}$ (availability per interval) and overall availability $\phi$ is expressed as $\phi =1-{\left(1-{p}_{a}\right)}^{j}$. This formula corresponds to the specific time when each entity is first detected. The point-specific ${p}_{{a}_{k}}$ is derived by summing up the time-interval specific probabilities ${p}_{{a}_{jk}}$ across time intervals $j=1, 2,\dots .,J$. This can be represented as ${p}_{{a}_{k}}=\sum_{j=1}^{J}{p}_{{a}_{jk}}$.

Finally, ${N}_{{pres}_{kt}}$ represents the proportion of individuals from the superpopulation present within the plot. We assumed a binomial distribution, expressed as ${N}_{{pres}_{kt}}\sim Binomial\left({p}_{p},{N}_{{super}_{k}}\right)$, where ${p}_{p}$ is the probability that each member of the superpopulation ${N}_{{super}_{k}}$ will be present during survey *t*^65,66^. The superpopulation was assumed to follow a Poisson distribution: ${N}_{{super}_{k}}\sim Poisson({\lambda }_{k})$. Further assumptions related to each component model are elaborated upon elsewhere (e.g.,^64,67^).

#### Simulation methods

We developed a simulation study to assess model performance under varying survey efforts, and differing probabilities of availability and presence. A few studies on point-count data inferences have indicated that model efficacy is influenced by factors such as detection probability, the expected number of individuals observed, and the intensity of survey efforts^18,51,63^. Many of these simulation studies primarily focused on understanding the model’s effectiveness for species that are moderately to highly available and commonly detected during point counts, but often, the species for which habitat is being managed may have lower availability and be detected less commonly during surveys. Consequently, our study employed diverse scenarios that encompassed a range of availability probabilities, densities, and survey efforts representing less common species.

Simulation scenarios reflected our survey methods. We used point count survey plots with a radius of 150 m, the four distance bins used in our field surveys (i.e., 0–25 m, > 25–50 m, > 50–100 m, and > 100–150 m), and the $J$ = 3 time sub-intervals used in our surveys (i.e., 0–3 min, 4–5 min, and 5–10 min). For the distribution $M\sim Poisson\left(\lambda \right)$, we set the value of $\lambda$ to 5. Both presence and availability probabilities were varied at three levels: 0.4, 0.6, and 0.8. We adopted a half-normal detection function with a scale parameter $\sigma$ = 55, which produced detection probabilities of 0.27. The number of bird point counts was examined at intervals of 50, 150, 300, and 500. Additionally, the number of repeated surveys, $T$, ranged from 1 to 3. These settings culminated in 76 unique simulation scenarios, each replicated 100 times.

To facilitate comparisons across all simulations, we assessed the relative bias and coverage probabilities for availability, presence, and mean abundance. All models were fit using jagsUI, which provides access to JAGS using Markov chain Monte Carlo (MCMC) algorithms to generate posterior distributions of the parameters in program R^68,69^^,^^70^. Three parallel chains were simulated for 650,000 iterations. The first 50,000 iterations served as burn-in, and 5000 were for adaptation. We thinned the chains by 20 to streamline model output. For all parameters, we employed vague priors: ${p}_{a}$ and ${p}_{p}$ were both set to $Beta\left(\text{1,1}\right)$, $log\left(\sigma \right)\sim Uniform(\text{0,10})$, and $\lambda \sim Gamma\left(\text{0.1,0.1}\right)$. Chain convergence, or stationarity, was evaluating using the Gelman-Rubin diagnostic statistics, combined with an examination of chain histories and the posterior density plots^71^.

### Bird surveys

We conducted annual point count surveys at Little River ($n$ = 30, range = 25–41), Caddo Lake ($n$ = 43, range = 25–64), and Little Sandy ($n$ = 18, range = 15–18) NWRs from 2008 to 2020, following methods aligned with Wilson and Twedt^9^ Between 15 May and 30 June, a single person surveyed each point. At Little Sandy, the same number of survey points were revisited annually, while at Little River and Caddo Lake, 25 core points were surveyed each year, with additional points drawn at Little River and Caddo Lake, which were revisited at least 2 or more years during the study period. We did not survey when it was raining or during strong winds. We surveyed for 10 min and recorded the distance and time for every bird observed or heard. Detections of birds were binned into distance intervals of 0–25 m, > 25–50 m, > 50–100 m, and > 100–150 m; and time sub-intervals of 0–3 min, > 3–5 min, and > 5–10 min. Any birds detected beyond the 150-m range or those merely flying over without using the forest were excluded from the analysis.

#### Density and trend estimates

We estimated avian species densities using the previously mentioned integrated model. This model was extended to account for time-specific variation related to the time series of data collected among refuges. In this expanded model, the sequence of counts from annual sampling occasion $t=1, 2,\dots .,T$ at each set of points $k=1, 2,\dots .,K$ within refuge $i=1, \dots ,I$ were denoted by $y$. The observed data, ${y}_{kt}$, were represented by the matrix $Y=\left\{{y}_{kt}:k=1, 2,\dots .,K;t=1, 2,\dots .,T\right\}$ and captured the number of individual birds observed during each survey. The number of birds seen, $h\left({y}_{kt+1}|{{y}_{kt},N}_{{avail}_{kt+1}},{p}_{{d}_{k}}\right)$, and the number of available, $f\left({N}_{{avail}_{kt+1}}|{y}_{kt},{N}_{kt+1},{\phi }_{k}\right)$, were both treated as binomial outcomes. The total abundance arises from a Poisson distribution, $g\left({N}_{kt+1}|{y}_{kt},{N}_{{avail}_{kt+1}},\rho ,\beta \right)$, modeled dynamically as a Markovian model. Initial processes were defined as ${y}_{k\rho }\sim Binomial\left({N}_{{avail}_{k\rho }},{p}_{{d}_{k}}\right)$, ${N}_{{avail}_{k\rho }}\sim Binomial\left({N}_{k\rho },{\phi }_{k}\right)$, and ${N}_{k\rho }\sim Poisson\left({\lambda }_{k\rho }\right),$ where ${\lambda }_{k\rho }\sim\Gamma \left({a}_{t-1},{b}_{t-1}\right)$. The subsequent processes were modeled as: ${y}_{kt}\sim Binomial\left({N}_{{avail}_{kt}},{p}_{{d}_{k}}\right)$ and ${N}_{{avail}_{kt}}\sim Binomial\left({N}_{kt},{\phi }_{k}\right)$. The terms ${p}_{{d}_{k}}$ and ${\phi }_{k}$ represent the combined probability of perception and probability of availability for point count $k$, as previously described. We let ${N}_{kt}$ follow a Poisson distribution, ${N}_{kt}\sim Poisson({\lambda }_{kt})$, where the mean or state variable ${\lambda }_{kt}$ of the distribution evolves according to a stationary autoregressive process of order $p$ (AR($p$)). The autocorrelation parameters of this process are denoted as ${\rho }_{i}$, $i=\text{1,2},..p$,

$$\lambda_{kt} = \mathop \sum \limits_{i = 1}^{p} \rho_{i} N_{kt - 1} + \left( {1 - \mathop \sum \limits_{i = 1}^{p} \rho_{i} } \right)exp\left( {X_{kt} \beta } \right)$$

where ${X}_{kt}$ represents a matrix of regressors that are exogenous of ${N}_{kt}$, $\forall i=\text{1,2},\dots p$. The vector $\beta$ consists of regression parameters. This includes a site nested within a refuge random effect, ${\delta }_{refuges}$, which accounts for the variation in mean abundance among different refuges. The instantaneous percentage change in the estimated ${N}_{kt}$ for a change was calculated to describe the long run percentage trend.

The detection probability was modeled as an intercept-only model via the parameter ${\sigma }_{k}$ through the equation:

$$log\left( {\sigma_{k} } \right) = \rho_{0k}$$

where ${\rho }_{0k}\sim N\left(0, 0.01\right)$ is a site-specific random exchangeable error term. Additionally, the probability of availability ${p}_{{a}_{k}}$ was associated with an intercept-only model using the logit link function, as:

$$logit\left( {p_{{a_{k} }} } \right) = \alpha_{0}$$

where ${\alpha }_{0}\sim N\left(0, 0.01\right)$. Finally, the mean abundance was related to a random effects model through a log link function, as:

$$log\left( {\lambda_{kt} } \right) = \beta_{0} + \delta_{refuges}$$

where ${\beta }_{0}\sim N(\text{0,0.01})$, ${\delta }_{refuges}\sim N(0,\tau )$, and $\tau \sim\Gamma (\text{0.001,0.001})$ is the precision.

Models were implemented using jagsUI to access JAGS using Markov chain Monte Carlo (MCMC) algorithms to generate posterior distributions of the parameters^68,69^. Uninformative, independent priors were chosen for the model parameters. Specifically, we considered $Normal\left(\mu =0, \sigma =0.01\right)$ for all regression parameters (the intercept and slope coefficients). Three parallel chains were simulated for 300,000 iterations. The first 50,000 iterations served as burn-in, and 5,000 were for adaptation. We thinned the chains by 20 to streamline model output. Chain convergence, or stationarity, was evaluating using the Gelman-Rubin diagnostic statistics, combined with an examination of chain histories and the posterior density plots^71^.

### Sample size estimation

We calculated the sample size and the requisite number of points needed to achieve a specified coefficient of variation for point count surveys $k$ for each species within each refuge annually. The encounter rate was determined as the average number of detections per survey point, calculated for each survey as ${n}_{0}/{k}_{0}$, with ${n}_{0}$ representing total detections and ${k}_{0}$ representing the number of points surveyed. Our aim was to achieve predetermined coefficients of variation (${CV}_{t}$) to suit different objectives: 0.15 for surveys with a research focus and 0.25 as a critical effect size for monitoring effects of management^15,16^. These thresholds correspond to levels of precision necessary for reliably detecting population trends and informing conservation and management decisions. To estimate the necessary number of sample points, we used the following formula:

$$k = \frac{q}{{\left( {CV_{t} \left( {\hat{D}} \right)} \right)^{2} }} \times \frac{{k_{0} }}{{n_{0} }}$$

where $\widehat{D}$ represents the estimated density. The value of $q$ was determined by multiplying ${n}_{0}$ by the square of the observed coefficient of variation for $\widehat{D}$^64^.

To understand the number of point counts used by other research in comparison to those employed in our current study, we searched the literature for studies on bird species in bottomland hardwood and riparian forests using Google Scholar. We employed a combination of keywords related to “point counts,” “bottomland hardwood and riparian forest birds,” “multispecies surveys,” and searches related to specific species. We built a comprehensive summary describing the typical range and methodologies of point counts used in the field. We focused on studies detailing the number of point counts or studies estimating the number of point counts needed to effectively survey a species or multiple species. This approach allowed us to compare our study’s methodology against existing literature and assess the adequacy and comparability of our point count data in the broader context of avian surveys in bottomland hardwood and riparian forest ecosystems.

## Supplementary Information

Supplementary Material 1.

## Data Availability

The data will be provided upon request to the corresponding author.
