# Supplementary material for: Assessing trends and density of bird species in bottomland hardwood forests and riparian forests using simulation and sample size optimization for surveys
Source: Sci Rep. 2025 Feb 28;15:7137. doi: 10.1038/s41598-025-91804-4 (PMC11871069; doi:10.1038/s41598-025-91804-4)
Supplement: Supplementary file 1 — Supplementary Material 1. [file 41598_2025_91804_MOESM1_ESM.docx]

Appendix. Estimated avian density and 95% credibility intervals (CI) for avian species in bottomland hardwood and riparian forest at Caddo Lake National Wildlife Refuge, Texas that were surveyed from 2008 to 2020. The numbers of detections (*n*) during 10-min point counts were used to estimate species-specific probability of detection ($p_{a}$), probability of detection ($p_{d}$), and autoregressive model of order 1 and 2 parameters ($\gamma$).

| Species | |  | Availability | |  | Detection | |  | AR(1) | | AR(2) | | | Density (birds/ km^2^) | |
| --- | --- | --- | --- | --- | --- | --- | --- | --- | --- | --- | --- | --- | --- | --- | --- |
| Common and scientific name | $n$ |  | $p_{a}$ | 95% CI |  | $p_{d}$ | 95% CI |  | $\gamma_{1}$ | 95% CI |  | $\gamma_{2}$ | 95% CI | D | 95% CI |
| Acadian Flycatcher (ACFL), *Empidonax virescens* | 101 |  | 0.86 | 0.76−0.93 |  | 0.24 | 0.21−0.29 |  | 0.97 | 0.92−0.99 |  | − | − | 13 | 10−17 |
| American Crow (AMCR), *Corvus brachyrhynchos* | 320 |  | 0.88 | 0.83−0.92 |  | 0.97 | 0.95−0.99 |  | 0.91 | 0.87−0.95 |  | 0.09 | 0.01−0.13 | 10 | 9−11 |
| Barred Owl (BADO), *Strix varia* | 16 |  | 0.77 | 0.62−0.94 |  | 0.98 | 0.86−0.99 |  | 0.96 | 0.90−0.99 |  | − | − | 1 | 0−2 |
| Black-and-white Warbler (BAWW), *Mniotilta varia* | 17 |  | 0.36 | 0.14−0.73 |  | 0.43 | 0.25−0.56 |  | 0.99 | 0.93−0.99 |  | − | − | 1 | 0−2 |
| Blue-gray Gnatcatcher (BGGN), *Polioptila caerulea* | 175 |  | 0.74 | 0.61−0.85 |  | 0.09 | 0.08−0.10 |  | 0.91 | 0.86−0.95 |  | 0.09 | 0.04−0.14 | 66 | 53−82 |
| Brown-headed Cowbird (BHCO), *Molothrus ater* | 69 |  | 0.20 | 0.14−0.35 |  | 0.44 | 0.35−0.56 |  | 0.96 | 0.91−0.99 |  | − | − | 26 | 13−41 |
| Brown-headed Nuthatch (BHNU), *Sitta pusilla* | 19 |  | 0.38 | 0.14−0.81 |  | 0.27 | 0.15−0.56 |  | 0.98 | 0.95−0.99 |  | − | − | 8 | 2−19 |
| Blue Jay (BLJA), *Cyanocitta cristata* | 175 |  | 0.30 | 0.14−0.52 |  | 0.56 | 0.47−0.65 |  | 0.91 | 0.87−0.95 |  | 0.08 | 0.05−0.13 | 40 | 19−76 |
| Carolina Chickadee (CACH), *Poecile carolinensis* | 161 |  | 0.51 | 0.30−0.69 |  | 0.24 | 0.22−0.27 |  | 0.90 | 0.81−0.99 |  | − | − | 44 | 30−74 |
| Carolina Wren (CARW), *Thryothorus ludovicianus* | 190 |  | 0.72 | 0.60−0.81 |  | 0.41 | 0.36−0.47 |  | 0.84 | 0.78−0.89 |  | 0.15 | 0.11−0.22 | 22 | 18−27 |
| Chipping Sparrow (CHSP), *Spizella passerina* | 43 |  | 0.39 | 0.06−0.75 |  | 0.54 | 0.50−0.56 |  | 0.96 | 0.90−0.99 |  | − | − | 5 | 1−19 |
| Common Yellowthroat (COYE), *Geothlypis trichas* | 15 |  | 0.65 | 0.17−0.96 |  | 0.50 | 0.28−0.91 |  | 0.97 | 0.92−0.99 |  | − | − | 2 | 0−6 |
| Downy Woodpecker (DOWO), *Picoides pubescens* | 29 |  | 0.32 | 0.14−0.67 |  | 0.52 | 0.35−0.56 |  | 0.81 | 0.20−0.99 |  | − | − | 6 | 2−13 |
| Eastern Wood-pewee (EAWP), *Contopus virens* | 91 |  | 0.78 | 0.63−0.88 |  | 0.79 | 0.61−0.99 |  | 0.94 | 0.86−0.99 |  | − | − | 5 | 4−7 |
| Great Crested Flycatcher (GCFL), *Myiarchus crinitus* | 11 |  | 0.34 | 0.14−0.79 |  | 0.49 | 0.26−0.99 |  | 0.89 | 0.11−0.99 |  | − | − | 4 | 1−9 |
| Hairy Woodpecker (HAWO), *Picoides villosus* | − |  | − | − |  | − | − |  | − | − |  | − | − | − | − |
| Hooded Warbler (HOWA), *Setophaga citrina* | 204 |  | 0.84 | 0.75−0.89 |  | 0.36 | 0.32−0.41 |  | 0.87 | 0.82−0.92 |  | 0.13 | 0.08−0.18 | 21 | 18−24 |
| Indigo Bunting (INBU), *Passerina cyanea* | 225 |  | 0.89 | 0.84−0.93 |  | 0.41 | 0.37−0.47 |  | 0.94 | 0.90−0.99 |  | − | − | 18 | 16−21 |
| Kentucky Warbler (KEWA), *Geothlypis formosa* | 95 |  | 0.55 | 0.34−0.74 |  | 0.42 | 0.35−0.51 |  | 0.77 | 0.69−0.85 |  | 0.23 | 0.15−0.31 | 13 | 8−20 |
| Louisiana Waterthrush (LOWA), *Parkesia motacilla* | − |  | − | − |  | − | − |  | − | − |  | − | − | − | − |
| Mourning Dove (MODO), *Zenaida macroura* | 107 |  | 0.76 | 0.61−0.87 |  | 0.96 | 0.94−0.99 |  | 0.92 | 0.83−0.99 |  | − | − | 5 | 4−6 |
| Northern Cardinal (NOCA), *Cardinalis cardinalis* | 453 |  | 0.63 | 0.53−0.72 |  | 0.44 | 0.40−0.48 |  | 0.87 | 0.83−0.90 |  | − | − | 51 | 43−62 |
| Northern Flicker (NOFL), *Colaptes auratus* | − |  | − | − |  | − | − |  | − | − |  | − | − | − | − |
| Northern Parula (NOPA), *Setophaga americana* | 20 |  | 0.74 | 0.30−0.96 |  | 0.53 | 0.36−0.56 |  | 0.98 | 0.94−0.99 |  | − | − | 2 | 1−4 |
| Pileated Woodpecker (PIWO), *Dryocopus pileatus* | 118 |  | 0.29 | 0.14−0.53 |  | 0.97 | 0.95−0.99 |  | 0.98 | 0.93−0.99 |  | − | − | 11 | 5−20 |
| Pine Warbler (PIWA), *Setophaga pinus* | 330 |  | 0.81 | 0.74−0.87 |  | 0.38 | 0.34−0.42 |  | 0.89 | 0.86−0.93 |  | 0.11 | 0.07−0.14 | 33 | 28−38 |
| Prairie Warbler (PRAW), *Setophaga discolor* | − |  | − | − |  | − | − |  | − | − |  | − | − | − | − |
| Prothonotary Warbler (PROW), *Protonotaria citrea* | 10 |  | 0.72 | 0.17−0.99 |  | 0.50 | 0.23−0.56 |  | 0.92 | 0.10−0.99 |  | − | − | 1 | 0−3 |
| Red-bellied Woodpecker (RBWO), *Melanerpes carolinus* | 174 |  | 0.62 | 0.42−0.77 |  | 0.57 | 0.49−0.68 |  | 0.89 | 0.84−0.94 |  | 0.11 | 0.06−0.16 | 15 | 11−22 |
| Red-eyed Vireo (REVI), *Vireo olivaceus* | 403 |  | 0.87 | 0.83−0.91 |  | 0.33 | 0.30−0.36 |  | 0.89 | 0.86−0.92 |  | − | − | 39 | 35−44 |
| Red-headed Woodpecker (RHWO), *Melanerpes erythrocephalus* | 174 |  | 0.54 | 0.17−0.81 |  | 0.73 | 0.52−0.99 |  | 0.84 | 0.77−0.94 |  | 0.16 | 0.06−0.23 | 6 | 3−15 |
| Red-shouldered Hawk (RSHA), *Buteo lineatus* | 57 |  | 0.65 | 0.19−0.92 |  | 0.98 | 0.89−0.99 |  | 0.98 | 0.92−0.99 |  | − | − | 2 | 1−4 |
| Summer Tanager (SUTA), *Piranga rubra* | 143 |  | 0.42 | 0.20−0.62 |  | 0.35 | 0.30−0.40 |  | 0.91 | 0.83−0.99 |  | − | − | 36 | 21−70 |
| Swainson’s Warbler (SWWA), *Limnothlypis swainsonii* | − |  | − | − |  | − | − |  | − | − |  | − | − | − | − |
| Tufted Titmouse (TUTI), *Baeolophus bicolor* | 301 |  | 0.80 | 0.72−0.86 |  | 0.32 | 0.29−0.36 |  | 0.87 | 0.83−0.90 |  | 0.13 | 0.10−0.17 | 35 | 30−41 |
| White-breasted Nuthatch (WBNU), *Sitta carolinensis* | 38 |  | 0.34 | 0.14−0.66 |  | 0.40 | 0.30−0.54 |  | 0.94 | 0.83−0.99 |  | − | − | 11 | 4−23 |
| White-eyed Vireo (WEVI), *Vireo griseus* | 291 |  | 0.84 | 0.77−0.89 |  | 0.29 | 0.26−0.32 |  | 0.79 | 0.72−0.85 |  | 0.21 | 0.15−0.28 | 36 | 31−42 |
| Wood Thrush (WOTH), *Hylocichla mustelina* | − |  | − | − |  | − | − |  | − | − |  | − | − | − | − |
| Yellow-breasted Chat (YBCH), *Icteria virens* | 270 |  | 0.87 | 0.81−0.91 |  | 0.56 | 0.50−0.64 |  | 0.93 | 0.90−0.97 |  | − | − | 18 | 16−21 |
| Yellow-billed Cuckoo (YBCU), *Coccyzus americanus* | 185 |  | 0.47 | 0.24−0.66 |  | 0.52 | 0.45−0.61 |  | 0.91 | 0.85−0.96 |  | − | − | 25 | 16−46 |
| Yellow-throated Vireo (YTVI), *Vireo flavifrons* | 16 |  | 0.37 | 0.14−0.78 |  | 0.38 | 0.26−0.57 |  | 0.64 | 0.15−0.96 |  | − | − | 6 | 2−14 |
| Yellow-throated Warbler (YTWA), *Setophaga dominica* | − |  | − | − |  | − | − |  | − | − |  | − | − | − | − |
|  |  |  |  |  |  |  |  |  |  |  |  |  |  |  |  |

Appendix. Estimated avian density and 95% credibility intervals (CI) for avian species in bottomland hardwood forest at Little River National Wildlife Refuge, Oklahoma that were surveyed from 2008 to 2020. The numbers of detections (*n*) during 10-min point counts were used to estimate species-specific probability of detection ($p_{a}$), probability of detection ($p_{d}$), and autoregressive model of order 1 and 2 parameters ($\gamma$).

| Species | |  | Availability | |  | Detection | |  | AR(1) | | AR(2) | | | Density (birds/ km^2^) | |
| --- | --- | --- | --- | --- | --- | --- | --- | --- | --- | --- | --- | --- | --- | --- | --- |
| Common and scientific name | $n$ |  | $p_{a}$ | 95% CI |  | $p_{d}$ | 95% CI |  | $\gamma_{1}$ | 95% CI |  | $\gamma_{2}$ | 95% CI | D | 95% CI |
| Acadian Flycatcher (ACFL), *Empidonax virescens* | 373 |  | 0.92 | 0.87−0.95 |  | 0.41 | 0.36−0.47 |  | 0.97 | 0.89−0.99 |  | − | − | 52 | 44−61 |
| American Crow (AMCR), *Corvus brachyrhynchos* | 71 |  | 0.75 | 0.39−0.92 |  | 0.94 | 0.92−0.99 |  | 82 | 0.64−0.96 |  | 0.18 | 0.04−0.36 | 5 | 4−10 |
| Barred Owl (BADO), *Strix varia* | 10 |  | 0.65 | 0.05−0.99 |  | 0.97 | 0.80−0.99 |  | 0.88 | 0.55−0.99 |  | − | − | 2 | 0−12 |
| Black-and-white Warbler (BAWW), *Mniotilta varia* | 24 |  | 0.37 | 0.04−0.83 |  | 0.35 | 0.21−0.63 |  | 0.48 | 0.03−0.99 |  | − | − | 19 | 3−75 |
| Blue-gray Gnatcatcher (BGGN), *Polioptila caerulea* | 158 |  | 0.86 | 0.74−0.93 |  | 0.20 | 0.17−0.24 |  | 0.97 | 0.87−0.99 |  | − | − | 48 | 38−62 |
| Brown-headed Cowbird (BHCO), *Molothrus ater* | 98 |  | 0.67 | 0.61−0.79 |  | 0.51 | 0.38−0.68 |  | 0.98 | 0.92−0.99 |  | − | − | 15 | 11−22 |
| Brown-headed Nuthatch (BHNU), *Sitta pusilla* | − |  | − | − |  | − | − |  | − | − |  | − | − | − | − |
| Blue Jay (BLJA), *Cyanocitta cristata* | 35 |  | 0.71 | 0.61−0.87 |  | 0.92 | 0.59−0.99 |  | 0.50 | 0.02−0.91 |  | − | − | 3 | 2−5 |
| Carolina Chickadee (CACH), *Poecile carolinensis* | 62 |  | 0.68 | 0.61−0.82 |  | 0.33 | 0.25−0.44 |  | 0.53 | 0.03−0.99 |  | − | − | 15 | 10−21 |
| Carolina Wren (CARW), *Thryothorus ludovicianus* | 296 |  | 0.76 | 0.63−0.85 |  | 0.98 | 0.86−0.99 |  | 0.75 | 0.56−0.90 |  | 0.25 | 0.10−0.44 | 21 | 18−26 |
| Chipping Sparrow (CHSP), *Spizella passerina* | − |  | − | − |  | − | − |  | − | − |  | − | − | − | − |
| Common Yellowthroat (COYE), *Geothlypis trichas* | 46 |  | 0.82 | 0.64−0.95 |  | 0.86 | 0.64−0.95 |  | 0.97 | 0.94−0.99 |  | − | − | 4 | 2−7 |
| Downy Woodpecker (DOWO), *Picoides pubescens* | 70 |  | 0.75 | 0.62−0.89 |  | 0.58 | 0.40−0.98 |  | 0.27 | 0.02−0.66 |  | 0.73 | 0.34−0.98 | 9 | 5−14 |
| Eastern Wood-pewee (EAWP), *Contopus virens* | 90 |  | 0.68 | 0.61−0.79 |  | 0.97 | 0.91−0.99 |  | 0.97 | 0.89−0.99 |  | − | − | 7 | 5−8 |
| Great Crested Flycatcher (GCFL), *Myiarchus crinitus* | 74 |  | 0.73 | 0.40−0.91 |  | 0.98 | 0.83−0.99 |  | 0.97 | 0.85−0.99 |  | − | − | 6 | 4−10 |
| Hairy Woodpecker (HAWO), *Picoides villosus* | − |  | − | − |  | − | − |  | − | − |  | − | − | − | − |
| Hooded Warbler (HOWA), *Setophaga citrina* | 108 |  | 0.70 | 0.61−0.83 |  | 0.96 | 0.75−0.99 |  | 0.98 | 0.94−0.99 |  | − | − | 8 | 6−12 |
| Indigo Bunting (INBU), *Passerina cyanea* | 107 |  | 0.80 | 0.60−0.92 |  | 0.98 | 0.88−0.99 |  | 0.96 | 0.94−0.99 |  | − | − | 7 | 5−10 |
| Kentucky Warbler (KEWA), *Geothlypis formosa* | 154 |  | 0.83 | 0.69−0.92 |  | 0.97 | 0.95−0.99 |  | 0.98 | 0.91−0.99 |  | − | − | 10 | 8−12 |
| Louisiana Waterthrush (LOWA), *Parkesia motacilla* | 13 |  | 0.78 | 0.62−0.96 |  | 0.96 | 0.75−0.99 |  | 0.87 | 0.33−0.99 |  | − | − | 1 | 0−2 |
| Mourning Dove (MODO), *Zenaida macroura* | − |  | − | − |  | − | − |  | − | − |  | − | − | − | − |
| Northern Cardinal (NOCA), *Cardinalis cardinalis* | 358 |  | 0.69 | 0.54−0.80 |  | 0.97 | 0.94−0.99 |  | 0.97 | 0.91−0.99 |  |  |  | 28 | 23−35 |
| Northern Flicker (NOFL), *Colaptes auratus* | − |  | − | − |  | − | − |  | − | − |  | − | − | − | − |
| Northern Parula (NOPA), *Setophaga americana* | 66 |  | 0.68 | 0.24−0.90 |  | 0.89 | 0.59−0.99 |  | 0.92 | 0.80−0.99 |  | − | − | 7 | 3−17 |
| Pileated Woodpecker (PIWO), *Dryocopus pileatus* | 60 |  | 0.45 | 0.15−0.81 |  | 0.97 | 0.93−0.99 |  | 0.92 | 0.47−0.99 |  | − | − | 9 | 4−22 |
| Pine Warbler (PIWA), *Setophaga pinus* | 57 |  | 0.72 | 0.26−0.92 |  | 0.54 | 0.36−0.90 |  | 0.98 | 0.96−0.99 |  | − | − | 9 | 4−24 |
| Prairie Warbler (PRAW), *Setophaga discolor* | 17 |  | 0.92 | 0.62−0.99 |  | 0.89 | 0.44−0.99 |  | 0.30 | 0.02−0.81 |  | 0.70 | 0.19−0.99 | 2 | 1−3 |
| Prothonotary Warbler (PROW), *Protonotaria citrea* | 141 |  | 0.54 | 0.16−0.78 |  | 0.98 | 0.87−0.99 |  | 0.94 | 0.13−0.99 |  | − | − | 16 | 9−46 |
| Red-bellied Woodpecker (RBWO), *Melanerpes carolinus* | 167 |  | 0.56 | 0.28−0.77 |  | 0.98 | 0.94−0.99 |  | 0.97 | 0.92−0.99 |  | − | − | 17 | 11−32 |
| Red-eyed Vireo (REVI), *Vireo olivaceus* | 537 |  | 0.87 | 0.82−0.91 |  | 0.68 | 0.60−0.78 |  | 0.98 | 0.92−0.99 |  | − | − | 47 | 40−56 |
| Red-headed Woodpecker (RHWO), *Melanerpes erythrocephalus* | 17 |  | 0.80 | 0.62−0.96 |  | 0.92 | 0.54−0.99 |  | 0.98 | 0.94−0.99 |  | − | − | 2 | 1−3 |
| Red-shouldered Hawk (RSHA), *Buteo lineatus* | 22 |  | 0.76 | 0.62−0.94 |  | 0.98 | 0.84−0.99 |  | 0.62 | 0.25−0.87 |  | − | − | 2 | 1−3 |
| Summer Tanager (SUTA), *Piranga rubra* | 136 |  | 0.55 | 0.18−0.79 |  | 0.85 | 0.60−0.99 |  | 0.95 | 0.81−0.99 |  | − | − | 18 | 10−47 |
| Swainson’s Warbler (SWWA), *Limnothlypis swainsonii* | 31 |  | 0.37 | 0.09−0.78 |  | 0.98 | 0.83−0.99 |  | 0.97 | 0.89−0.99 |  | − | − | 7 | 2−19 |
| Tufted Titmouse (TUTI), *Baeolophus bicolor* | 383 |  | 0.68 | 0.54−0.79 |  | 0.95 | 0.81−0.99 |  | 0.97 | 0.91−0.99 |  | − | − | 31 | 25−40 |
| White-breasted Nuthatch (WBNU), *Sitta carolinensis* | 45 |  | 0.51 | 0.15−0.86 |  | 0.69 | 0.39−0.99 |  | 0.49 | 0.03−0.98 |  | − | − | 9 | 3−27 |
| White-eyed Vireo (WEVI), *Vireo griseus* | 385 |  | 0.86 | 0.79−0.91 |  | 0.61 | 0.53−0.72 |  | 0.98 | 0.96−0.99 |  | − | − | 38 | 32−46 |
| Wood Thrush (WOTH), *Hylocichla mustelina* | − |  | − | − |  | − | − |  | − | − |  | − | − | − | − |
| Yellow-breasted Chat (YBCH), *Icteria virens* | 120 |  | 0.86 | 0.73−0.94 |  | 0.99 | 0.94−0.99 |  | 0.98 | 0.97−0.99 |  | − | − | 7 | 5−9 |
| Yellow-billed Cuckoo (YBCU), *Coccyzus americanus* | 232 |  | 0.38 | 0.15−0.62 |  | 0.98 | 0.96−0.99 |  | 0.96 | 0.85−0.99 |  | − | − | 37 | 20−79 |
| Yellow-throated Vireo (YTVI), *Vireo flavifrons* | 103 |  | 0.71 | 0.39−0.88 |  | 0.75 | 0.52−0.99 |  | 0.91 | 0.35−0.99 |  | − | − | 11 | 7−21 |
| Yellow-throated Warbler (YTWA), *Setophaga dominica* | 105 |  | 0.66 | 0.35−0.85 |  | 0.97 | 0.92−0.99 |  | 0.98 | 0.95−0.99 |  | − | − | 9 | 6−16 |
|  |  |  |  |  |  |  |  |  |  |  |  |  |  |  |  |

Appendix. Estimated avian density and 95% credibility intervals (CI) for avian species in bottomland hardwood forest at Little Sandy National Wildlife Refuge, Texas that were surveyed from 2008 to 2020. The numbers of detections (*n*) during 10-min point counts were used to estimate species-specific probability of detection ($p_{a}$), probability of detection ($p_{d}$), and autoregressive model of order 1 and 2 parameters ($\gamma$).

| Species | |  | Availability | |  | Detection | |  | AR(1) | | AR(2) | | | Density (birds/ km^2^) | |
| --- | --- | --- | --- | --- | --- | --- | --- | --- | --- | --- | --- | --- | --- | --- | --- |
| Common and scientific name | $n$ |  | $p_{a}$ | 95% CI |  | $p_{d}$ | 95% CI |  | $\gamma_{1}$ | 95% CI |  | $\gamma_{2}$ | 95% CI | D | 95% CI |
| Acadian Flycatcher (ACFL), *Empidonax virescens* | 194 |  | 0.96 | 0.93−0.98 |  | 0.52 | 0.42−0.64 |  | 0.86 | 0.39−0.99 |  | − | − | 54 | 42−69 |
| American Crow (AMCR), *Corvus brachyrhynchos* | 83 |  | 0.83 | 0.55−0.95 |  | 0.98 | 0.96−0.99 |  | 0.94 | 0.83−0.99 |  | − | − | 15 | 12−21 |
| Barred Owl (BADO), *Strix varia* | 22 |  | 0.82 | 0.26−0.99 |  | 0.98 | 0.88−0.99 |  | 0.74 | 0.04−0.99 |  | − | − | 4 | 2−12 |
| Black-and-white Warbler (BAWW), *Mniotilta varia* | − |  | − | − |  | − | − |  | − | − |  | − | − | − | − |
| Blue-gray Gnatcatcher (BGGN), *Polioptila caerulea* | 96 |  | 0.95 | 0.93−0.99 |  | 0.14 | 0.11−0.18 |  | 0.37 | 0.02−0.87 |  | 0.63 | 0.13−0.98 | 46 | 44−47 |
| Brown-headed Cowbird (BHCO), *Molothrus ater* | 17 |  | 0.82 | 0.62−0.98 |  | 0.27 | 0.15−0.49 |  | 0.60 | 0.02−0.99 |  | − | − | 12 | 5−24 |
| Brown-headed Nuthatch (BHNU), *Sitta pusilla* | − |  | − | − |  | − | − |  | − | − |  | − | − | − | − |
| Blue Jay (BLJA), *Cyanocitta cristata* | 25 |  | 0.79 | 0.62−0.96 |  | 0.97 | 0.78−0.99 |  | 0.98 | 0.89−0.99 |  | − | − | 5 | 3−7 |
| Carolina Chickadee (CACH), *Poecile carolinensis* | 91 |  | 0.70 | 0.61−0.84 |  | 0.26 | 0.20−0.33 |  | 0.43 | 0.01−0.99 |  | − | − | 71 | 48−99 |
| Carolina Wren (CARW), *Thryothorus ludovicianus* | 252 |  | 0.96 | 0.93−0.98 |  | 0.96 | 0.81−0.99 |  | 0.77 | 0.11−0.99 |  | − | − | 37 | 35−45 |
| Chipping Sparrow (CHSP), *Spizella passerina* | − |  | − | − |  | − | − |  | − | − |  | − | − | − | − |
| Common Yellowthroat (COYE), *Geothlypis trichas* | − |  | − | − |  | − | − |  | − | − |  | − | − | − | − |
| Downy Woodpecker (DOWO), *Picoides pubescens* | 57 |  | 0.42 | 0.15−0.78 |  | 0.62 | 0.41−0.99 |  | 0.94 | 0.74−0.99 |  | − | − | 39 | 14−95 |
| Eastern Wood-pewee (EAWP), *Contopus virens* | 54 |  | 0.64 | 0.24−0.89 |  | 0.49 | 0.34−0.74 |  | 0.99 | 0.96−0.99 |  | − | − | 27 | 13−66 |
| Great Crested Flycatcher (GCFL), *Myiarchus crinitus* | − |  | − | − |  | − | − |  | − | − |  | − | − | − | − |
| Hairy Woodpecker (HAWO), *Picoides villosus* | − |  | − | − |  | − | − |  | − | − |  | − | − | − | − |
| Hooded Warbler (HOWA), *Setophaga citrina* | − |  | − | − |  | − | − |  | − | − |  | − | − | − | − |
| Indigo Bunting (INBU), *Passerina cyanea* | 140 |  | 0.94 | 0.89−0.98 |  | 0.99 | 0.93−0.99 |  | 0.88 | 0.26−0.99 |  | − | − | 20 | 19−23 |
| Kentucky Warbler (KEWA), *Geothlypis formosa* | − |  | − | − |  | − | − |  | − | − |  | − | − | − | − |
| Louisiana Waterthrush (LOWA), *Parkesia motacilla* | − |  | − | − |  | − | − |  | − | − |  | − | − | − | − |
| Mourning Dove (MODO), *Zenaida macroura* | − |  | − | − |  | − | − |  | − | − |  | − | − | − | − |
| Northern Cardinal (NOCA), *Cardinalis cardinalis* | 255 |  | 0.98 | 0.92−0.99 |  | 0.31 | 0.28−0.36 |  | 0.28 | 0.01−0.89 |  | 0.72 | 0.11−0.99 | 112 | 95−131 |
| Northern Flicker (NOFL), *Colaptes auratus* | − |  | − | − |  | − | − |  | − | − |  | − | − | − | − |
| Northern Parula (NOPA), *Setophaga americana* | 115 |  | 0.92 | 0.84−0.97 |  | 0.47 | 0.37−0.61 |  | 0.98 | 0.90−0.99 |  | − | − | 37 | 25−48 |
| Pileated Woodpecker (PIWO), *Dryocopus pileatus* | 85 |  | 0.70 | 0.61−0.83 |  | 0.98 | 0.92−0.99 |  | 0.48 | 0.02−0.99 |  | − | − | 17 | 13−20 |
| Pine Warbler (PIWA), *Setophaga pinus* | 36 |  | 0.97 | 0.87−0.99 |  | 0.54 | 0.43−0.56 |  | 0.98 | 0.93−0.99 |  | − | − | 10 | 7−13 |
| Prairie Warbler (PRAW), *Setophaga discolor* | − |  | − | − |  | − | − |  | − | − |  | − | − | − | − |
| Prothonotary Warbler (PROW), *Protonotaria citrea* | 81 |  | 0.91 | 0.80−0.97 |  | 0.60 | 0.42−0.99 |  | 0.98 | 0.97−0.99 |  | − | − | 21 | 12−31 |
| Red-bellied Woodpecker (RBWO), *Melanerpes carolinus* | 117 |  | 0.58 | 0.23−0.82 |  | 0.97 | 0.93−0.99 |  | 0.61 | 0.02−0.99 |  | − | − | 31 | 19−71 |
| Red-eyed Vireo (REVI), *Vireo olivaceus* | 235 |  | 0.98 | 0.97−0.99 |  | 0.31 | 0.27−0.36 |  | 0.87 | 0.18−0.99 |  | − | − | 106 | 87−123 |
| Red-headed Woodpecker (RHWO), *Melanerpes erythrocephalus* | 19 |  | 0.87 | 0.44−0.99 |  | 0.39 | 0.21−0.83 |  | 0.97 | 0.87−0.99 |  | − | − | 9 | 3−19 |
| Red-shouldered Hawk (RSHA), *Buteo lineatus* | 16 |  | 0.71 | 0.18−0.99 |  | 0.98 | 0.87−0.99 |  | 0.37 | 0.01−0.89 |  | 0.63 | 0.11−0.99 | 4 | 2−13 |
| Summer Tanager (SUTA), *Piranga rubra* | 96 |  | 0.82 | 0.62−0.93 |  | 0.55 | 0.41−0.76 |  | 0.39 | 0.01−0.75 |  | 0.61 | 0.25−0.99 | 30 | 20−44 |
| Swainson’s Warbler (SWWA), *Limnothlypis swainsonii* | − |  | − | − |  | − | − |  | − | − |  | − | − | − | − |
| Tufted Titmouse (TUTI), *Baeolophus bicolor* | 231 |  | 0.91 | 0.85−0.95 |  | 0.48 | 0.39−0.58 |  | 0.55 | 0.13−0.97 |  | − | − | 73 | 57−91 |
| White-breasted Nuthatch (WBNU), *Sitta carolinensis* | 29 |  | 0.72 | 0.61−0.89 |  | 0.53 | 0.34−0.56 |  | 0.83 | 0.55−0.99 |  | − | − | 11 | 7−18 |
| White-eyed Vireo (WEVI), *Vireo griseus* | 122 |  | 0.94 | 0.87−0.98 |  | 0.49 | 0.39−0.63 |  | 0.94 | 0.80−0.99 |  | − | − | 37 | 27−48 |
| Wood Thrush (WOTH), *Hylocichla mustelina* | − |  | − | − |  | − | − |  | − | − |  | − | − | − | − |
| Yellow-breasted Chat (YBCH), *Icteria virens* | − |  | − | − |  | − | − |  | − | − |  | − | − | − | − |
| Yellow-billed Cuckoo (YBCU), *Coccyzus americanus* | 124 |  | 0.65 | 0.61−0.74 |  | 0.98 | 0.97−0.99 |  | 0.22 | 0.01−0.62 |  | 0.78 | 0.38−0.99 | 26 | 22−30 |
| Yellow-throated Vireo (YTVI), *Vireo flavifrons* | 78 |  | 0.79 | 0.63−0.92 |  | 0.41 | 0.31−0.55 |  | 0.42 | 0.03−0.96 |  | − | − | 34 | 22−50 |
| Yellow-throated Warbler (YTWA), *Setophaga dominica* | 86 |  | 0.88 | 0.74−0.96 |  | 0.83 | 0.55−0.99 |  | 0.46 | 0.02−0.97 |  | − | − | 17 | 12−25 |
|  |  |  |  |  |  |  |  |  |  |  |  |  |  |  |  |


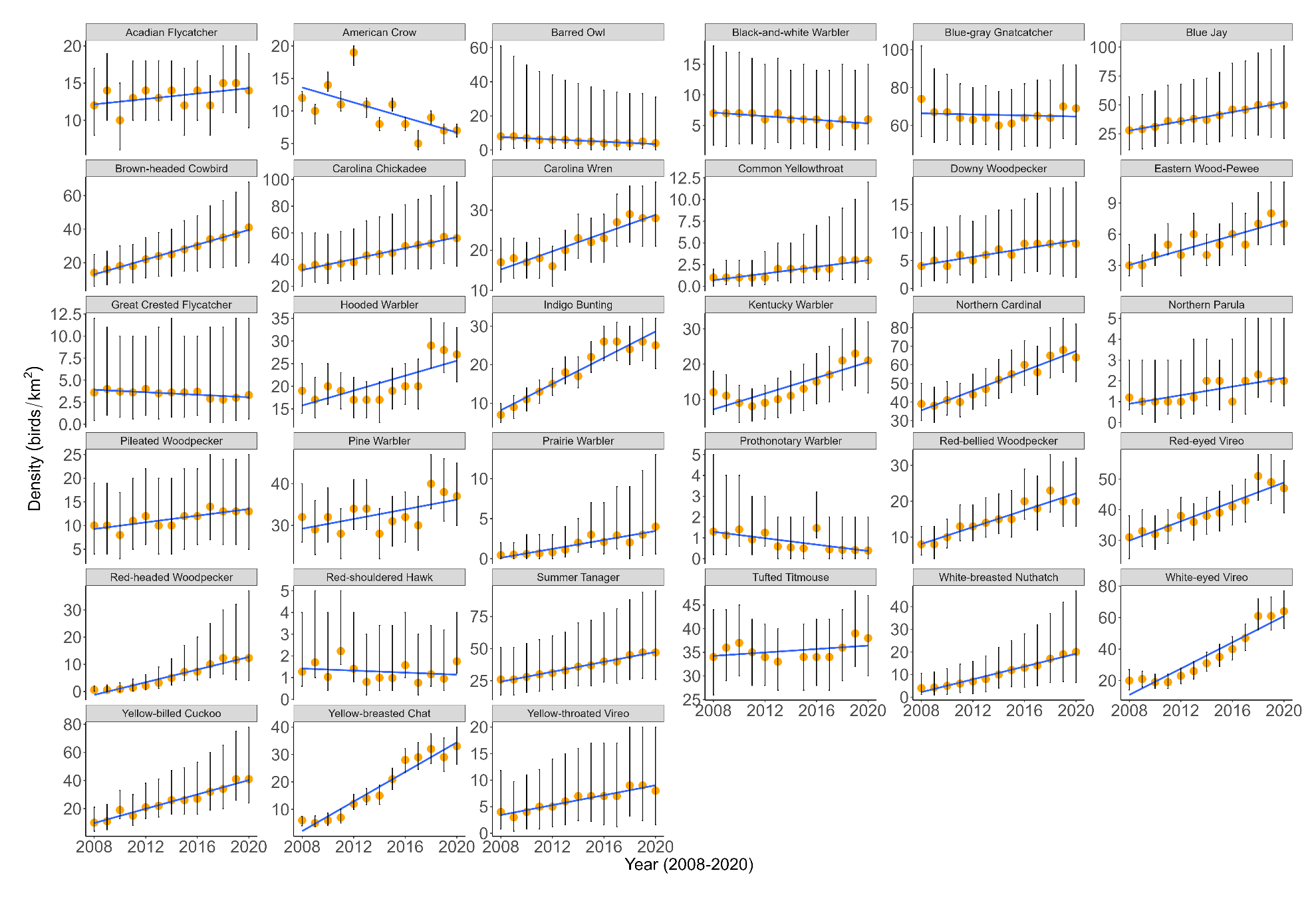
Appendix: Density estimates (birds per km^2^) for 33 bird species predicted from a refuge-specific (Caddo Lake National Wildlife Refuge) hybrid, time-to-detection hierarchical Bayesian model accounting for temporal variability. For each bird species, solid lines represent the mean density predictions, while the error bars denote the 95% credibility intervals on these posterior distributions. The closed circles correspond to annual, species-specific density estimates. Each panel corresponds to a different species. There scientific names are reported in Table 2.


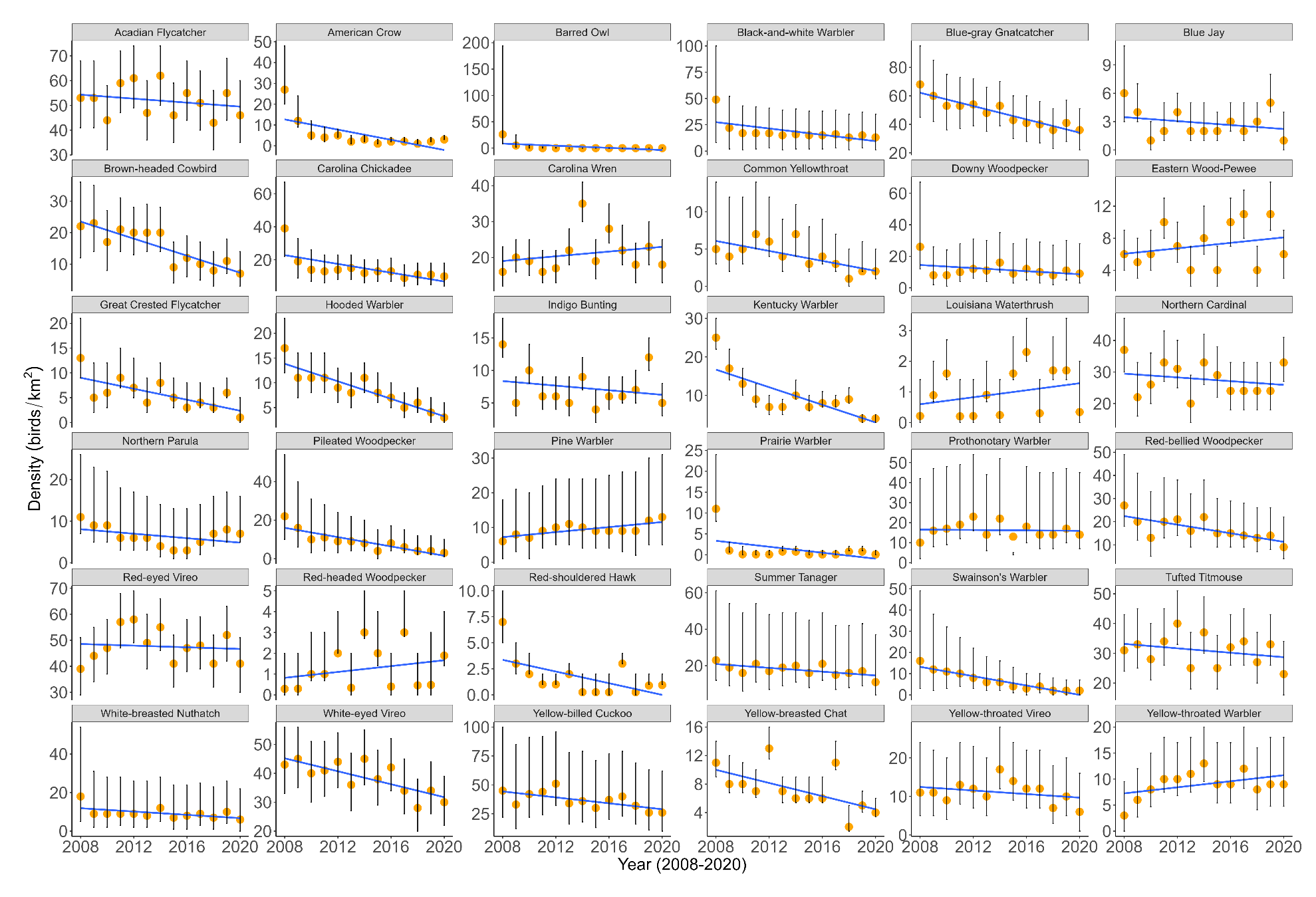


Appendix: Density estimates (birds per km^2^) for 36 bird species predicted from a refuge-specific (Little River National Wildlife Refuge) hybrid, time-to-detection hierarchical Bayesian model accounting for temporal variability. For each bird species, solid lines represent the mean density predictions, while the error bars denote the 95% credibility intervals on these posterior distributions. The closed circles correspond to annual, species-specific density estimates. Each panel corresponds to a different species. There scientific names are reported in Table 2.


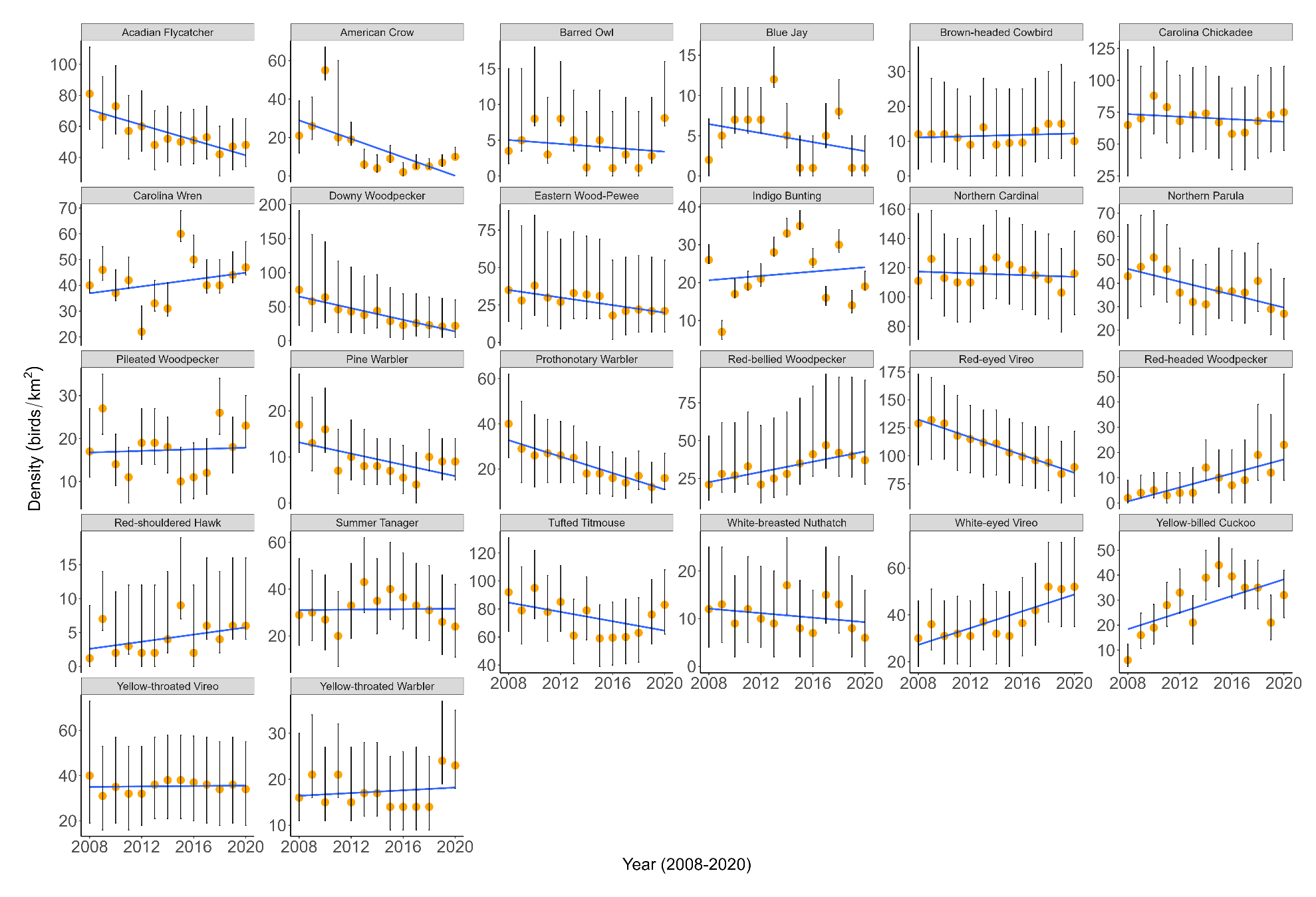


Appendix: Density estimates (birds per km^2^) for 30 bird species predicted from a refuge-specific (Little Sandy National Wildlife Refuge) hybrid, time-to-detection hierarchical Bayesian model accounting for temporal variability. For each bird species, solid lines represent the mean density predictions, while the error bars denote the 95% credibility intervals on these posterior distributions. The closed circles correspond to annual, species-specific density estimates. Each panel corresponds to a different species. There scientific names are reported in Table 2.
